# Stable, active CO_2_ reduction to formate via redox-modulated stabilization of active sites

**DOI:** 10.1038/s41467-021-25573-9

**Published:** 2021-09-01

**Authors:** Le Li, Adnan Ozden, Shuyi Guo, F. Pelayo García de Arquer, Chuanhao Wang, Mingzhe Zhang, Jin Zhang, Haoyang Jiang, Wei Wang, Hao Dong, David Sinton, Edward H. Sargent, Miao Zhong

**Affiliations:** 1 College of Engineering and Applied Sciences, National Laboratory of Solid State Microstructures, Collaborative Innovation Center of Advanced Microstructure, Jiangsu Key Laboratory of Artificial Functional Materials, Nanjing University, Nanjing, China; 2 Department of Mechanical and Industrial Engineering, University of Toronto, Toronto, Ontario Canada; 3 Kuang Yaming Honors School & Institute for Brain Sciences, Nanjing University, Nanjing, China; 4 Department of Electrical and Computer Engineering, University of Toronto, Toronto, Ontario Canada

**Keywords:** Electrocatalysis, Electrocatalysis

## Abstract

Electrochemical reduction of CO_2_ (CO_2_R) to formic acid upgrades waste CO_2_; however, up to now, chemical and structural changes to the electrocatalyst have often led to the deterioration of performance over time. Here, we find that alloying p-block elements with differing electronegativities modulates the redox potential of active sites and stabilizes them throughout extended CO_2_R operation. Active Sn-Bi/SnO_2_ surfaces formed in situ on homogeneously alloyed Bi_0.1_Sn crystals stabilize the CO_2_R-to-formate pathway over 2400 h (100 days) of continuous operation at a current density of 100 mA cm^−2^. This performance is accompanied by a Faradaic efficiency of 95% and an overpotential of ~ −0.65 V. Operating experimental studies as well as computational investigations show that the stabilized active sites offer near-optimal binding energy to the key formate intermediate *OCHO. Using a cation-exchange membrane electrode assembly device, we demonstrate the stable production of concentrated HCOO^–^ solution (3.4 molar, 15 wt%) over 100 h.

## Abstract (web summary)

Stable electrochemical reduction to formate is still challenging. Here, the authors demonstrate a redox-modulation and active-site stabilization strategy for CO_2_ to formate conversion over 100 days of continuous operation at 100 mA/cm^2^ with a cathodic energy efficiency of 70%.

## Introduction

Electrochemical CO_2_ reduction (CO_2_R) driven by electrical energy converts CO_2_ into low carbon footprint chemicals and fuels^1^. Among CO_2_R products, formic acid (HCOOH) or formate (HCOO^–^) is used in pharmaceutical, electrolytic metallurgy, leather, and fuel cell applications^2–6^. HCOO^–^ has a high market value per energy (cents/kWh)^7^, making it a candidate of particular interest.

Conventionally, HCOO^–^ is produced from the hydrolysis of methyl formate, which uses CH_3_OH and CO as starting materials. This chemical reforming process requires strict reaction conditions and high energy input^8–10^. In contrast, CO_2_R requires only CO_2_, water, and electrical energy^7,11,12^. Unfortunately, to date, CO_2_R catalysts and systems favouring HCOO^–^ have not achieved the required combination of high selectivity (Faradaic efficiency (FE))^13^, high reaction rate (current density)^13^, high energy efficiency (EE)^14^, and, in particular, long-term stability.

Among electrocatalytic materials studied, Sn is a promising candidate owing to its low cost and planetary abundance^15,16^. Sn has strong binding energy for *OCHO, and this favours the first-step CO_2_ hydrogenation in CO_2_-to-formate conversion^17,18^. The second step electron transfer requires high energy to reduce *OCHO to HCOO^–^, resulting in a large overpotential and consequently a low EE. Sn has medium binding energy to *COOH and *H, which makes it difficult to fully suppress CO and H_2_ generation; the FE for HCOO^–^ has therefore been limited to 80–85%. Recent studies of Sn-based core-shell structures, including Ag-Sn^19^, Cu-Sn^20^, Bi-Sn^21^, and phase-segregated bimetallic systems^22,23^, showed improved FE for HCOO^–^. Further improvement of the HCOO^–^ production rate and cathodic energy efficiency (CEE) relative to current benchmarks (Supplementary Table 4) requires precise control of the elemental distributions in the active sites. In particular, knowledge of the electrochemical stability of Sn and Sn-based materials in aqueous electrolytes at different pH is lacking; indeed, among reported formate catalysts, it has been challenging to combine optimal adsorption energetics for intermediate binding with sites stable against reconstruction^13–16^.

Here, we present active Sn-Bi/SnO_2_ surfaces that are grown conformally on uniformly alloyed Bi_0.1_Sn crystals. These surfaces support the stable reduction of CO_2_ to formate over a period in excess of 2400 h (100 days) of continuous operation, with a near-unity formate FE of over 95% and a CEE exceeding 70% at a current density of 100 mA cm^−2^ in 1 M potassium bicarbonate (KHCO_3_) and potassium hydroxide (KOH) electrolytes at pH 11. This stability and CEE is significantly improved compared to literature benchmarks^13,24–27^. The selectivity and energy efficiency obtained to meet the performance required for a positive net present value in an economic analysis of formate production^11^. Computational studies reveal that, across a wide range (1.5–12.5%) of Bi incorporation into Sn, the facets of Sn-Bi alloys and Sn-Bi/SnO_2_ composites provide sites with near-optimal binding energy to *OCHO. This approach lowers the reaction energy in CO_2_-to-formate conversion. The by-products CO and H_2_ are suppressed due to their increased reaction energy on Sn-Bi alloys. The redox-modulated Sn-Bi/SnO_2_ surfaces remain active and protect Bi_0.1_Sn catalysts against corrosion or reconstruction during extended CO_2_R. We further demonstrate stable production of 3.4 molar (15 wt%) HCOO^–^ (formate) solution of over 100 h at a constant current density of 60 mA cm^−2^ with an average full-cell EE of ~27% in a membrane electrode assembly (MEA) system based on a cation-exchange membrane (CEM), highlighting that Bi_0.1_Sn catalysts are reliable for long-lasting HCOO^–^ (formate) production in different CO_2_R systems.

## Results

### Characterization

**Fig. 1:**
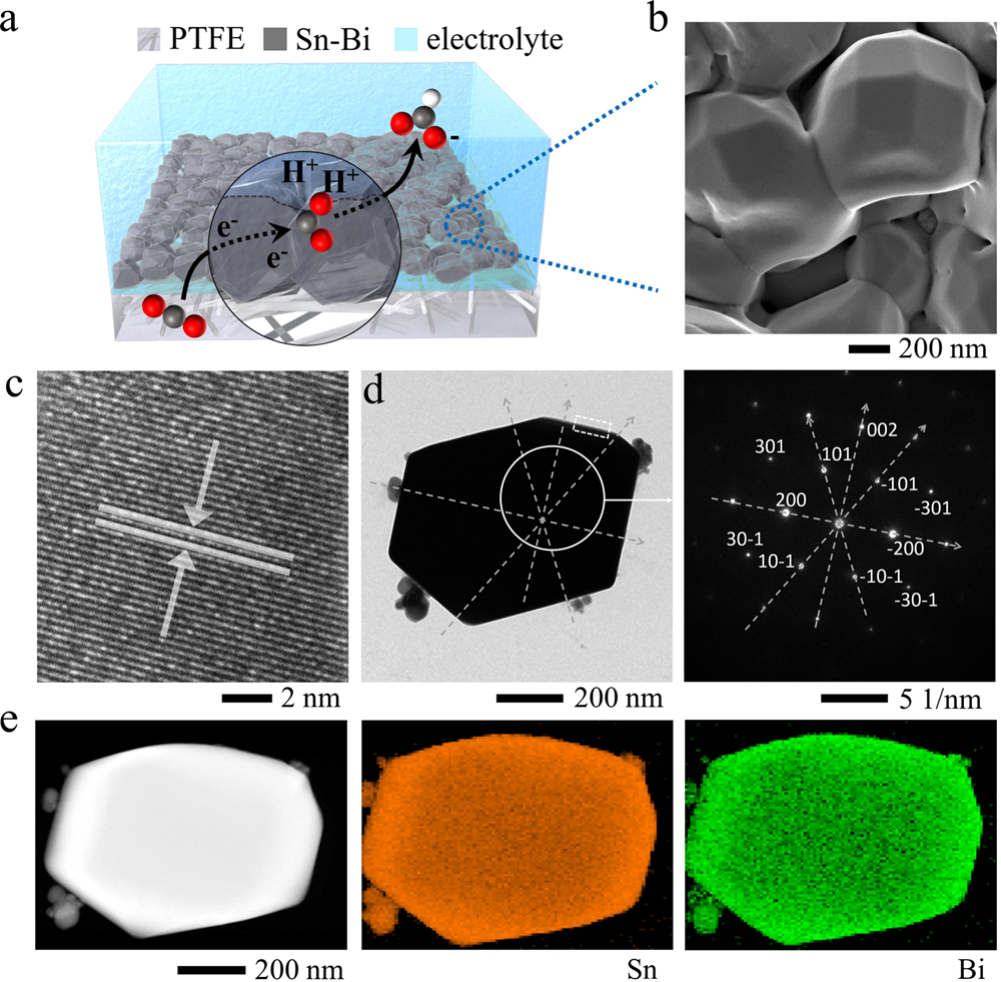
Structural and elemental analyses of Bi0.1Sn electrocatalysts. **a** Schematic of Bi_0.1_Sn electrocatalyst on a polytetrafluoroethylene gas diffusion layer for electroreduction of CO_2_. **b** SEM, **c** HRTEM, **d** TEM, SAED, and, **e** STEM, EDS analyses of Bi_0.1_Sn electrocatalyst. The high-resolution TEM image in (**c**) shows the lattice fringes corresponding to the Sn (200) facet. The dashed box in (**d**) is the location to take the HRTEM.

We used thermal evaporation to produce Bi_*x*_Sn (*x* = 0.1, 0.2, 0.3), Bi, and Sn precatalysts on polytetrafluoroethylene (PTFE) gas diffusion substrates (Supplementary Fig. 1). Dense and compact particle-film layers were formed for all Bi_*x*_Sn (*x* = 0.1, 0.2, 0.3), Bi, and Sn catalysts, ensuring electrical conductivity for CO_2_R (Supplementary Fig. 2). The particle size is 200–300 nm for the Bi and 1–1.5 μm for the Sn catalyst. Bi_0.2_Sn and Bi_0.3_Sn possess similar particle sizes of 1–1.5 μm to that of Sn, but they fail to form a uniform alloy; Bi precipitates over a large area (Supplementary Figs. 3, 4). Bi_0.1_Sn forms uniform crystals with particle sizes of 700–1000 nm, as confirmed by scanning electron microscopy (SEM), transmission electron microscopy (TEM) images, and scanning transmission electron microscopy–energy dispersive spectroscopy (STEM-EDS) elemental mapping in Fig. 1b, c, e. The selected area electron diffraction (SAED) pattern of Bi_0.1_Sn exhibits distinct single-crystal diffraction spots corresponding to the Sn plane indexes (Fig. 1d), suggesting that Bi is incorporated into the Sn crystals. The facets exposed are primarily (200) and (101), consistent with the observations from high-resolution TEM analysis (Fig. 1c). We carried out STEM-EDS, SEM-EDS, and XPS analyses, and obtained similar Bi concentrations of ~10 at.% for the Bi_0.1_Sn catalyst (Supplementary Figs. 5–9 and Table 1), suggesting that Bi and Sn are uniformly distributed from the surface to the bulk.

**Table 1:** The molar concentration of Bi and Sn in the Bi_0.1_Sn obtained by XPS, SEM-EDS, and TEM-EDS analyses.

	TEM-EDX	SEM-EDX	XPS
Bi: Sn (at.%)	~0.15:1	~0.1:1	~0.12:1

### Density functional theory (DFT) calculations

**Fig. 2:**
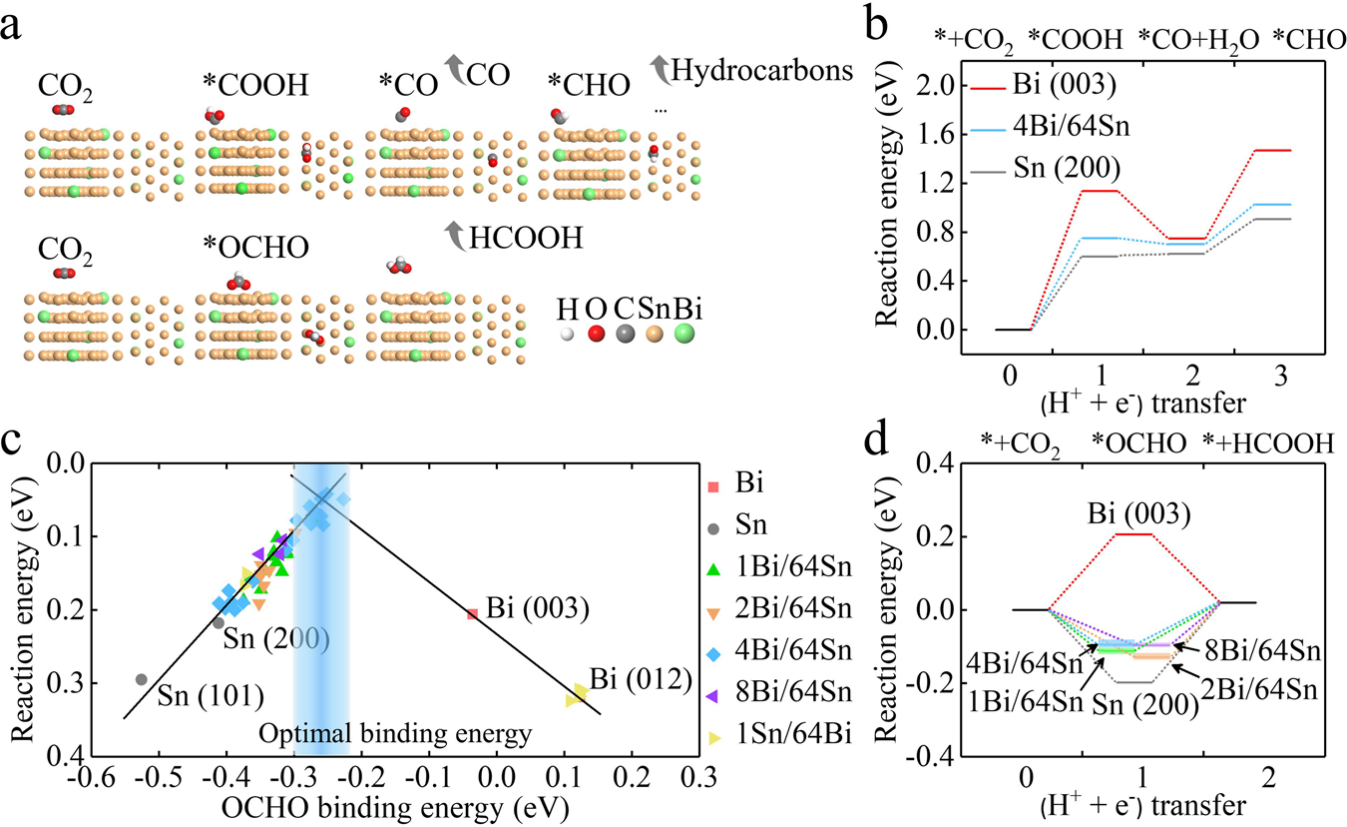
DFT calculations. **a** Possible pathways of electrochemical CO_2_ reduction. The top panel shows the *COOH pathway to produce CO, CH_4_, or C_2+ _hydrocarbons. The bottom panel shows the *OCHO pathway to produce HCOO^–^. Bi, Sn, C, O, and H atoms are represented by green, yellow, grey, red, and white spheres, respectively. **b** Reaction energy for CO formation on Bi (003), Sn (200), and Bi_4_Sn_64_ (200) surfaces without applying any external potential (*U* = 0 eV). **c** The volcano plot describing *OCHO adsorption energy versus CO_2_-to-HCOO^–^ reaction energy. **d** Reaction energy for HCOO^–^ formation on Bi (003), Sn (200), and Bi_*y*_Sn_64_ (*y* = 1, 2, 4, 8) surfaces without applying any external potential (*U* = 0 eV).

In light of the above characterizations, we built models and performed DFT studies to calculate the reaction energy on Bi, Sn, and Bi_*y*_Sn_64_ (*y* = 1, 2, 4, 8) surfaces (details in Supplementary Figs. 10–15 and Supplementary Table 1). As shown in Fig. 2a, the production of CO, CH_4_, and other C_2+ _hydrocarbons generally proceeds via the *COOH intermediate pathway^28,29^. *COOH adsorption requires the outer shell electrons of catalysts to have both energy that matches the LUMO energy of CO_2_, and electronic orbitals that overlap with molecular orbitals of the C–O π bond. Production of HCOO^–^ often occurs via the *OCHO intermediate pathway^14,18^. This requires catalysts to possess suitable binding energy to O. Bi and Sn are both p-block metals that, as shown in Fig. 2b, d, display significantly uphill reaction energy to *COOH compared to that to *OCHO, indicating that HCOO^–^ is the major product.

Since the intermediate *OCHO is energetically more favourable than *COOH on Bi, Sn, and Bi_0.1_Sn surfaces, we examined their reaction energy for HCOO^–^ production in detail. Figure 2d reveals that Bi (003) tends to bind *OCHO too weakly, that Sn (002) may bind *OCHO too strongly, and that of the calculated BiSn slabs, Bi_4_Sn_64_ and Bi_8_Sn_64_ show the optimal binding energy to *OCHO of near to –0.13 eV, indicating low reaction energy for the CO_2_-to-HCOO^–^ conversion. We plotted the reaction energy of different catalyst archetypes as a function of their adsorption energy to *OCHO at 298 K and 1 atm. (Fig. 2c). We took an average of the binding energy on enumerated possible configurations and present the averaged binding energy in Fig. 2d. Bi_*y*_Sn_64_ (*y* = 1, 2, 4, 8), with a relatively wide range of Bi ratios in different Sn facets, exhibits an abundance of adsorption sites with improved *OCHO binding energy that enhances CO_2_ reduction (DFT calculation details are given in Supplementary Figs. 12–15). The volcano relationship predicts that Bi_4_Sn_64_ (incorporation of 6.25% Bi into Sn) may be the most active (Supplementary Table 2). We further find that Bi_4_Sn_64_ elevates the reaction energy to CO and H_2_, suppressing the generation of CO, H_2_, and C_2+ _hydrocarbons (Fig. 2b and Supplementary Fig. 11). In summary, our computational simulations point to the alloying of Sn with Bi as a strategy to enhance HCOO^–^ production.

### CO_2_R in aqueous flow cells

**Fig. 3:**
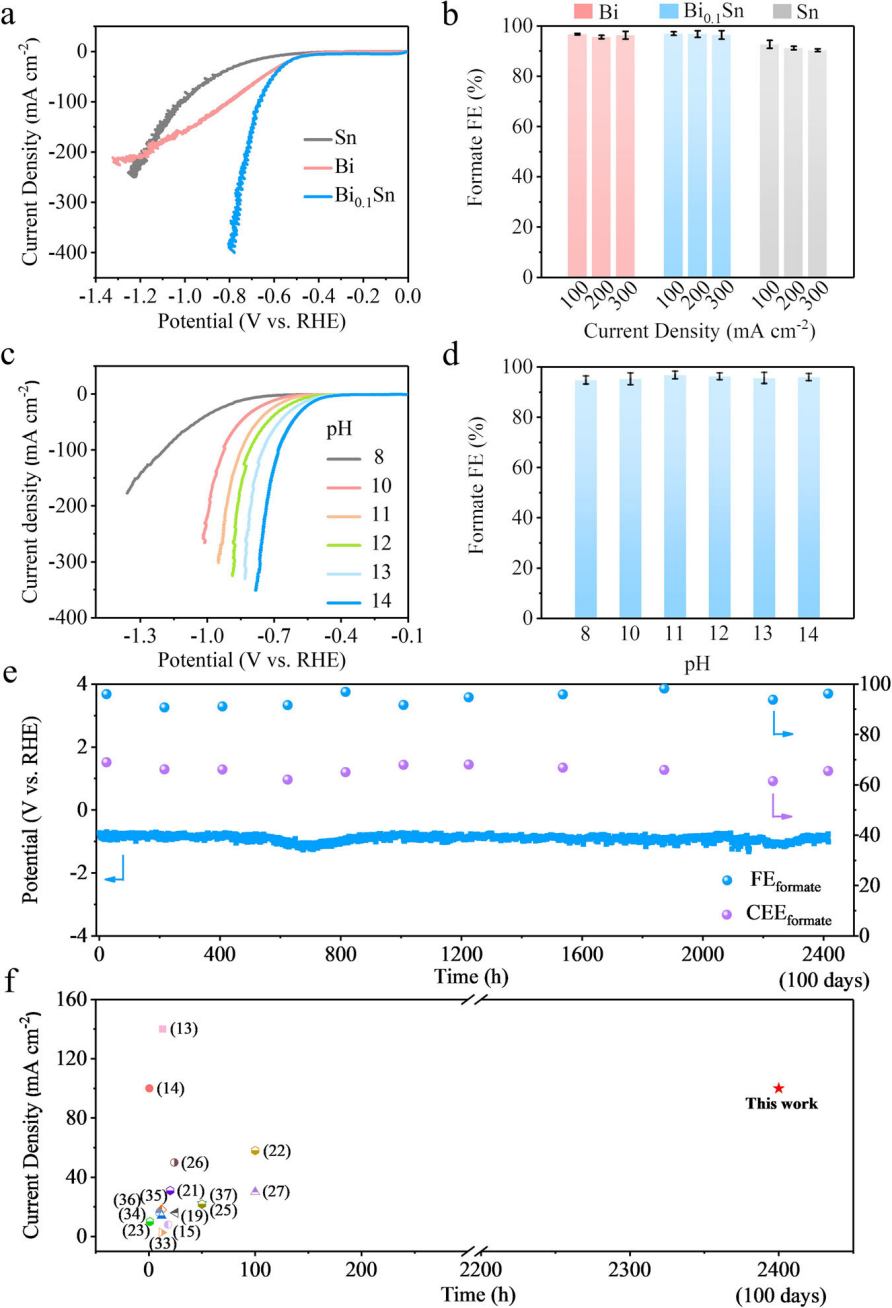
CO2 electroreduction performance using Bi0.1Sn, Bi, and Sn catalysts. **a** Linear sweep voltammetry curves of Bi_0.1_Sn, Bi, and Sn in 1 M KOH electrolyte. **b** The corresponding formate FEs under different current densities. **c** Linear sweep voltammetry curves. **d** The corresponding formate FEs in 1 M KHCO_3_ and KOH electrolytes at different pH (pH = 8, 10, 11, 12, 13, and 14) at a current density of 100 mA cm^−2^. **e** The CO_2_-reduction chronopotentiometry curve (blue line), CO_2_-to-formate FE (blue dots), and CO_2_-to-formate half-cell energy conversion efficiency (purple dots) in 1 M KHCO_3_ and KOH electrolyte at pH = 11 at an applied current density of 100 mA cm^−2^. **f** Comparison of our work with previously published data. Error bars correspond to the standard deviation of five independent measurements.

The CO_2_R performance of Bi_*x*_Sn alloys (*x* = 0.1, 0.2, 0.3), as well as pure Bi or Sn control samples, was first evaluated in 1 M KOH electrolyte (pH = 14) in a flow-cell electrolyser. Ag/AgCl electrodes were used as the reference electrodes, and commercial Ni foams were used as the anodes for the water oxidation reaction in a three-electrode setup. Linear sweep voltammetry (LSV) curves obtained from 0 V to –2 V vs. reversible hydrogen electrode (V_RHE_) show that Bi_0.1_Sn has the most positive onset potential for CO_2_R (Fig. 3a and Supplementary Fig. 16a). This is consistent with the DFT calculations that incorporating 1.5–12.5% Bi into Sn lowers the reaction energy in the electrochemical CO_2_-to-formate conversion (Fig. 2). A rapid increase of the cathodic current is observed with Bi_0.1_Sn at potentials more negative than –0.5 V_RHE_. To study the electrochemical characteristics of the Bi_0.1_Sn, Bi, and Sn catalysts, we carried out the electrochemical active surface area (ECSA), and electrochemical impedance spectroscopy (EIS) analyses^30^. The estimated ECSAs of Bi and Bi_0.1_Sn are twice as large as that of Sn (Supplementary Fig. 17). Also, Bi_0.1_Sn has the lowest interface resistance (Supplementary Fig. 18). These results confirm that Bi_0.1_Sn reduces the potential loss and, consequently, improves the CEE.

To quantify the FEs of CO_2_R products, Bi_*x*_Sn (*x* = 0.1, 0.2, 0.3), Bi, and Sn catalysts were evaluated in a chronopotentiometry mode by applying current densities of 100, 200, and 300 mA cm^−2^. Gaseous and liquid products were quantified by gas chromatography (GC) and nuclear magnetic resonance (NMR), respectively. As shown in Fig. 3b and Supplementary Fig. 16b, the Bi_0.1_Sn catalyst shows over 95% FE for HCOO^–^ in a wide range of current densities. Only a small amount of H_2_ and CO was detected, and no other liquid products were formed (Supplementary Figs. 19, 20). A CO_2_-to-formate CEE of ~75% was achieved at pH 14 at 100 mA cm^−2^. Under similar conditions, Bi_0.2_Sn, Bi_0.3_Sn, and Bi catalysts produce 95% FE for HCOO^–^, but they require a more negative potential for the same current density (Supplementary Fig. 16b, 21 and Supplementary Table 3). The Sn catalyst produces an FE of ~85% for HCOO^–^, along with a constant total FE of ~15% for H_2_ and CO. It also requires a more negative potential to reach 100 mA cm^−2^, and thus the CEE for HCOO^–^ is limited to 50%.

To assess the stability of the Bi_0.1_Sn catalyst during CO_2_R, carbon nanoparticles (NPs) and graphite were coated on the Bi_0.1_Sn/PTFE electrode to form a hybrid structure of graphite/carbon NPs/Bi_0.1_Sn/PTFE. This structure enables uniform distribution of the electrolyte and electrical current over the Bi_0.1_Sn surfaces^31,32^. Note that the coated carbon nanoparticles and graphite are not CO_2_R electrocatalysts and they don’t produce any formate under our CO_2_R operating conditions. Importantly, we coated carbon nanoparticles and graphite for all the Bi, Sn, and Bi_0.1_Sn catalysts, comparing their CO_2_R activity under the same working conditions. We are therefore certain that the performance obtained solely represents the properties of each catalyst. We also kept the electrolyte pH unchanged during the long-term CO_2_R stability test so that the CO_2_R results are all compared under the same pH conditions. In our studies, Bi_0.1_Sn catalyst produces HCOO^–^ with an FE exceeding 95% for over 170 h at 100 mA cm^−2^ and an overpotential of ~–0.5 V_RHE_ in 1 M KOH electrolyte (Supplementary Fig. 22). Control experiments for Bi and Sn samples using similar carbon/graphite coatings led to both lower CEEs and worse stability within 50–70 h (see Supplementary Methods and Supplementary Figs. 23–25).

To evaluate the effect of pH on the activity and stability of CO_2_R, we studied the performance of Bi_0.1_Sn catalysts in KHCO_3_ and KOH electrolytes at different pH levels (see the Supplementary Methods for details). As shown in Supplementary Fig. 26, the formate FEs are above 90% at all pH (8–14) at current densities from 25 to 300 mA cm^−2^. This indicates that the formate FE is directly related to the properties of catalysts because the CO_2_R kinetics are more favourable than those of the competing hydrogen evolution reaction under these working conditions. The use of alkaline electrolytes also shifts the working potential positively with the increase of electrolyte pH. As shown in Figs. 3c, 3d and Supplementary Fig. 27, the CEEs for HCOO^–^ are above 70% at 100 mA cm^−2^ at pH greater than 11. We performed 100-hour stability tests at varying pH (11–14) at a current density of 100 mA cm^−2^ and analysed their surface morphology, composition, and other material properties after the reaction by SEM, XRD, XPS, and EDX (Supplementary Figs. 28–31). At pH 11, the morphology change is minor compared to those at higher pH levels after 100 h of operation. This is in line with the Pourbaix diagram (Supplementary Fig. 32), in which both metallic Bi and Sn are cathodically protected at potentials more negative than –0.35 V_RHE_ at pH 11. The self-corrosion potentials of Bi_0.1_Sn catalysts at pH 11–14 in Supplementary Fig. 34 indicate that, thermodynamically, the tendency of corrosion increases with the increase of pH.

Remarkably, we achieved efficient CO_2_R over 2400 h (100 days) at pH 11 with stable 70% CEE (Fig. 3e). A full-cell EE of 35% (without *IR* correction) was obtained using unmodified, commercial Ni foams as anodes for the water oxidation reaction in a flow-cell system (Supplementary Fig. 35). This is significant in developing CO_2_R electrolysers that meet the long operational stability requirements for commercialization^12^ of at least 1000 h with current densities exceeding 100 mA cm^−2^. Ours is the first demonstration of CO_2_R stability achieving this goal. To put our results in a broader context, we plotted our data with reported values^13–15,19,21–23,25–27,33–37^ in Fig. 3f. The stability and CEE at current densities ≥ 100 mA cm^−2^ we achieved outperform literature benchmarks by two orders of magnitude, indicating a critical milestone in the field of CO_2_R field.

We compared the CO_2_R performance of Bi and Sn at 100 mA cm^−2^ and in 1 M KHCO_3_ and KOH electrolytes at pH 11. Sn produces ~80% FE for HCOO^–^ with a constant total FE of ~20% for H_2_ and CO. The hydrogen evolution reaction increases and HCOO^–^ decreases throughout electrolysis (Supplementary Fig. 37). Bi produces 95% FE for HCOO^–^, but the CEE is much lower (Supplementary Figs. 36, 37). We found that the stability of Bi was poor: We performed 10 independent tests for Bi samples in an aqueous system with a continuous operation of 100 h. We observed performance decay and peeling off of Bi in all tests (Supplementary Fig. 38).

**Fig. 4:**
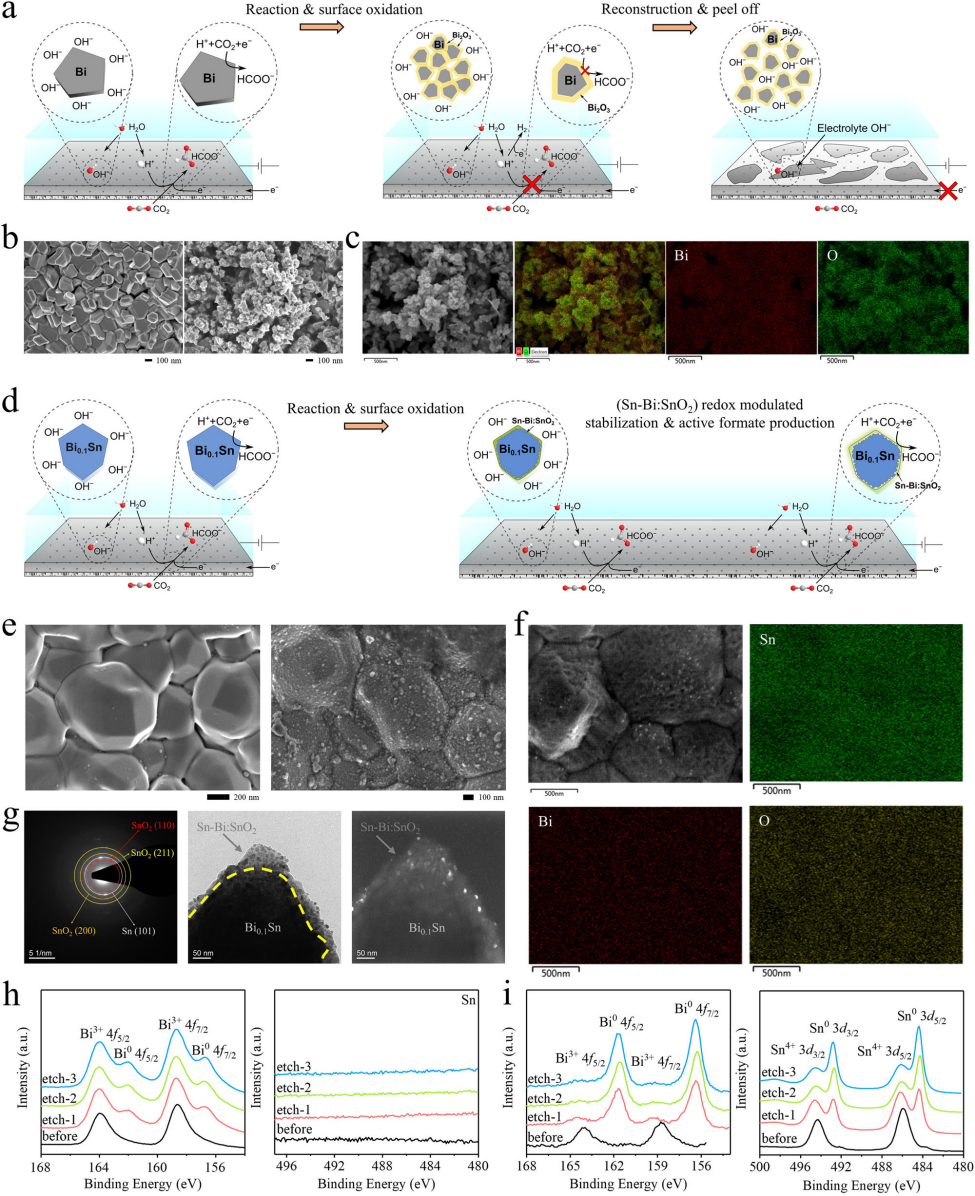
Structural and elemental analyses of the Bi and Bi0.1Sn electrocatalyst before and after CO2 reduction at pH 11 and a current density of 100 mA cm^-2^. **a** Schematic of Bi reconstruction during the electrochemical reaction. **b** SEM results of the as-prepared Bi nanoplates, and the reconstructed Bi/Bi_2_O_3_ core/shell nanoparticles after 70 h of reaction. **c** SEM-EDX elemental mapping results of the Bi/Bi_2_O_3_ catalysts after 70 h of reaction. **d** Schematic of a redox-modulated Bi_0.1_Sn/SnO_2_ surface on Bi_0.1_Sn during the electrochemical reaction. **e** SEM results of the as-prepared Bi_0.1_Sn crystals, and the (Bi_0.1_Sn/SnO_2_)/Bi_0.1_Sn particles over 100 h of reaction. **f** SEM-EDX elemental mapping results of the (Sn-Bi/SnO_2_)/Bi_0.1_Sn catalyst over 100 h of reaction. **g** The SAED pattern, bright-field and dark-field TEM images of the Sn-Bi/SnO_2_ catalysts over 100 h of reaction. **h** XPS depth profiles of the post-reaction Bi after 50-h CO_2_ reduction at 100 mA cm^−2^ in 1 M KHCO_3_ and KOH electrolytes at pH = 11. **i** XPS depth profile of the post-reaction Bi_0.1_Sn after 100-h CO_2_ reduction at 100 mA cm^−2^ in 1 M KHCO_3_ and KOH electrolytes at pH = 11. The black, red, green and blue lines represent the XPS data before etching (black) and after different time courses of soft Ar etching (red, green, blue), respectively. The etched depth in each soft Ar etching is ~3–5 nm.

To diagnose the origins of catalyst instability during CO_2_R, we compared Bi and Bi_0.1_Sn catalysts before and after the reactions. After the 70-h test, the original 200–500 nm Bi crystals were completely reconstructed to 20–50 nm nanoparticles with a large amount of O on the surfaces (Fig. 4a–c). SEM-EDX and HRTEM analyses reveal that polycrystalline Bi_2_O_3_ was formed on Bi, which is in line with the XRD results for Bi after the reaction; the yellow line in the HRTEM image indicates the boundary between the Bi bulk and the Bi_2_O_3_ surface (Fig. 4b, c and Supplementary Figs. 39–40). This chemical and structural transformation is associated with effects involving (i) the non-uniform distribution of electrical potential on the surfaces during the reaction^38,39^ and (ii) the existence of OH^–^ near Bi at pH 11 to make redox reactions between Bi and Bi_2_O_3_ continuously occur. Metastable Bi failed to form equilibrated phases on the surfaces and, thus, Bi was continuously reconstructed during the reaction. The reconstructed Bi_2_O_3_/Bi nanoparticles show increased electrical resistance to decrease the overall performance. As shown in the XPS depth profile studies, Bi^3+^ was detected on the 50h-reaction Bi throughout three rounds of the 3–5 nm soft Ar etching (Fig. 4h). This gives evidence that Bi^3+^ is presented in the bulk of the 50h-reaction Bi sample. In contrast, when we studied the evaporated Bi catalyst before reaction, Bi^3+^ disappeared after one round of ~3–5 nm soft Ar etching, indicating that the top surface Bi was oxidized in air and no bulk oxidation was observed (Supplementary Fig. 41). DFT studies in Supplementary Fig. 42 reveal that the Bi_2_O_3_ (002) and (210) facets largely shift the *OCHO reaction energy to ~–0.83 and ~0.30 eV, which is either too strong or too weak for the reaction as predicted by the volcano relationship in Fig. 2c. Taking these together with SEM, TEM, and electrochemical analyses for the Bi control samples before and after the reaction in Fig. 4b, c and Supplementary Figs. 38, 39, we conclude that Bi reconstructed to Bi_2_O_3_/Bi during the reduction, which leads to performance degradation and eventually catalyst peeling off.

In contrast, the Bi_0.1_Sn catalyst remains stable during the long CO_2_R operation under the same working conditions. After our tests, Bi_0.1_Sn remains intact on PTFE with morphology preserved (Fig. 4d–f and Supplementary Fig. 43). Dark-field and bright-field TEM images with SAED patterns and HRTEM visualized conformal ~20–50 nm SnO_2_/Sn-Bi mixture layers on Bi_0.1_Sn surfaces (Fig. 4g and Supplementary Figs. 44–46). The SAED patterns show a polycrystalline nature of surface SnO_2_, in line with the XRD results (Supplementary Figs. 47, 44c). We carried out calculations of different initial configurations with O involved in the Sn-Bi system on the surface (Supplementary Fig. 48). The results reveal that the homogeneously alloyed Bi_0.1_Sn crystal with few O atoms involved on the surfaces shows similar catalytic performance to that of the metallic Bi_0.1_Sn crystal. DFT studies also reveal that the SnO_2_ (110) facet remains active for HCOO^–^ production with the optimal binding energy of ~–0.15 eV for *OCHO (Supplementary Fig. 42).

As shown in the XPS depth profiles (Fig. 4i and Supplementary Fig. 49), after a round of ~ 3–5 nm soft Ar etching, Bi^0^, Sn^0^ and Sn^4+^ were observed in the consequent three rounds of the 3–5 nm soft Ar etching. The electrochemical redox potentials of Bi/Bi^3+^ and Sn/Sn^4+^ are

1 $${{{{{{\rm{Bi}}}}}}}_{2}{{{{{{\rm{O}}}}}}}_{3}+3{{{{{{\rm{H}}}}}}}_{2}{{{{{\rm{O}}}}}}+6{{{{{\rm{e}}}}}}\,\rightleftharpoons\, \,2{{{{{\rm{Bi}}}}}}+6{{{{{{\rm{OH}}}}}}}^{-}\,-0.46\,{{{{{\rm{V}}}}}}$$

2 $${{{{{{\rm{SnO}}}}}}}_{2}+2{{{{{{\rm{H}}}}}}}_{2}{{{{{\rm{O}}}}}}+4{{{{{\rm{e}}}}}}\,\rightleftharpoons\, {{{{{\rm{Sn}}}}}}+4{{{{{{\rm{OH}}}}}}}^{-}\,-0.945\,{{{{{\rm{V}}}}}}$$

This suggests that Sn should be oxidized before Bi. A redox-modulated balance between Sn and SnO_2_ was formed on the bimetallic active sites on the surface during CO_2_R. The redox modulation between Sn/Sn^4+^ protects the active BiSn:SnO_2_ against corrosion. We, therefore, suggest that we in situ formed immobilized, conformal and active BiSn:SnO_2_ surfaces during CO_2_ reduction, being stable against chemical and structural change throughout extended CO_2_R reaction over 2400 h of continuous operation.

After 2400 h, we observed densely packed Bi_0.1_Sn particles over the electrode with clear surface Bi and Sn signals identified by XPS (Supplementary Fig. 50). No significant leaching of Bi and Sn into solution was detected via inductively coupled plasma atomic emission spectroscopy (ICP-AES) analysis (Supplementary Fig. 51). We also provide SEM images and performance curves of Bi_0.1_Sn and Bi after different times taken from the stability tests as shown in Supplementary Fig. 52. We witness no peeling of the catalyst during the 2400 h test.

Note that a stable and efficient CO_2_R device requires stable and efficient reactions of CO_2_R and water oxidation. On the cathode side, Bi_0.1_Sn catalysts deliver excellent CO_2_R stability and activity via redox-modulated stabilization of active sites. On the anode side, it is vital to improving the stability and efficiency of the catalysts.

### CO_2_R in MEA systems

**Fig. 5:**
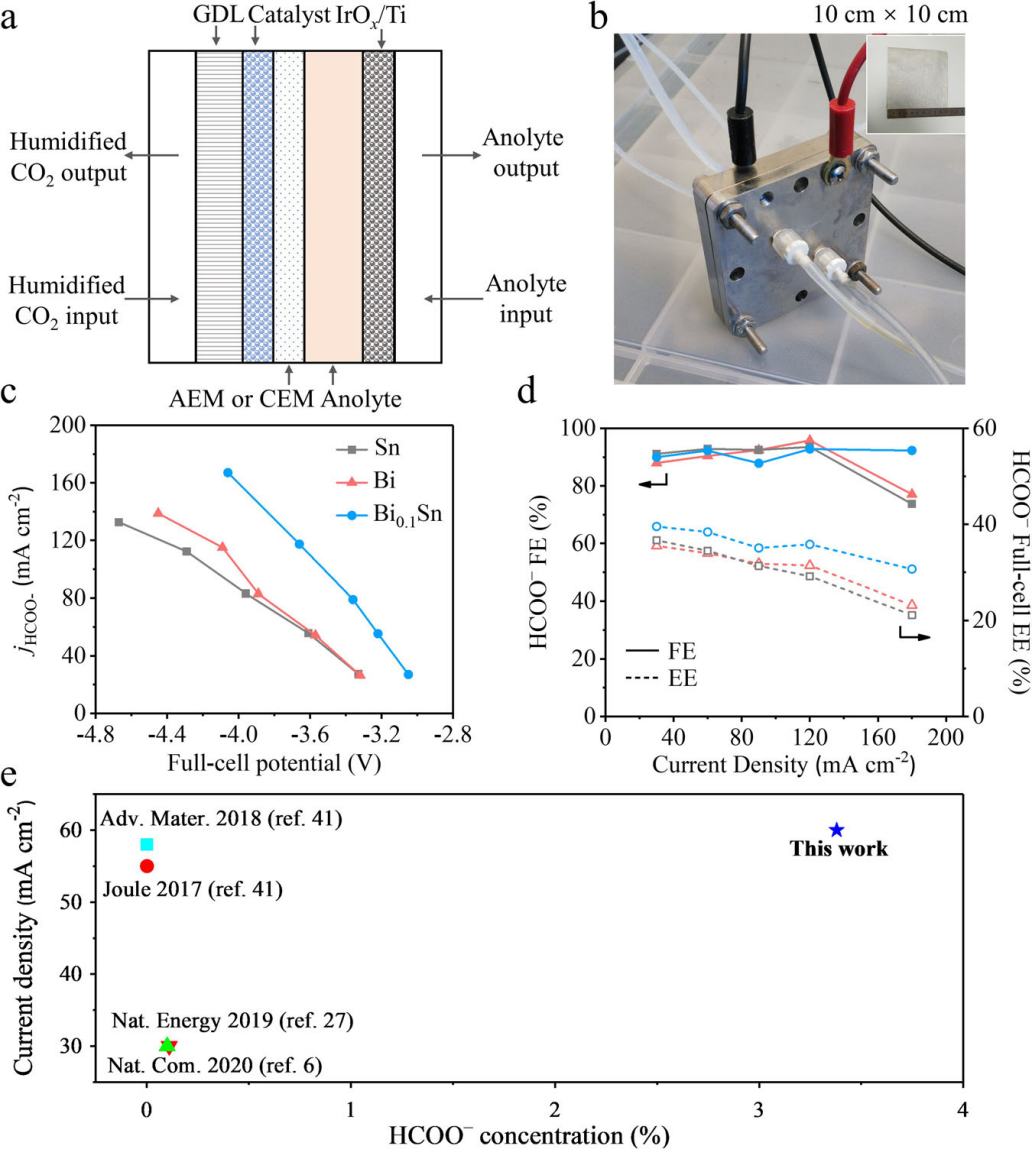
CO_2_ electroreduction performance in anion-exchange membrane (AEM)-based MEA systems. **a** Schematic of the MEA configuration. **b** The optical image of an MEA device. **c** Current densities vs. applied potentials of Bi, Sn, and Bi_0.1_Sn in AEM-based MEA systems. **d** HCOO^–^ FEs and EEs of Bi, Sn, and Bi_0.1_Sn at different current densities in AEM-based MEA systems. **e** Comparison of our data with literature benchmarks (We plot the concentration values obtained in the longest duration tests in each report).

To produce concentrated HCOO^–^ solutions, we assessed the CO_2_R performance of Bi, Sn, and Bi_0.1_Sn catalysts in an MEA system (Fig. 5a, b). We first used an anion-exchange membrane (AEM) to test the MEA performance. As shown in Fig. 5c, d, and Supplementary Table 5, Bi_0.1_Sn produced over 90% FE for HCOO^–^ in a wide range of current densities from 30 to 180 mA cm^−2^. A peak FE for HCOO^–^ of 97.8% was achieved at a current density of 120 mA cm^−2^ with a full-cell potential (without IR correction) of –3.6 V and a full-cell EE of 36%. As control experiments, pristine Bi and Sn delivered peak HCOO^–^ FEs of 95.8% and 93.5% at the same current density of 120 mA cm^−2^, but they required more negative potentials of –4.1 V and –4.3 V. As a result, the full-cell EEs for the MEAs using Bi and Sn catalysts were both lower, 31% and 29%, respectively. LSV curves confirmed the CO_2_R performance of Bi_0.1_Sn surpassed those of Bi and Sn in AEM-based MEA (Supplementary Fig. 53).

We then sought to translate the champion performance of Bi_0.1_Sn to a CEM-based MEA electrolyser as a strategy to minimize the crossover of HCOO^–^ (formate) to the anode side to producing a concentrated HCOO^–^ solution at the cathodic stream (HCOO^–^: 3.4 molar, 15 wt%). Replacing AEM with CEM likely changed the local pH near the catalyst surfaces during the reaction, leading to a shift of peak HCOO^–^ FE to relatively lower current densities^7,40^. By using a high anolyte flow rate of 15 mL/min., we obtained a peak HCOO^–^ FE of ~90% at 60 mA cm^−2^ with Bi_0.1_Sn (Supplementary Fig. 54 and Supplementary Table 5). We then optimized the single-pass flow rate of anolyte (0.1 M KHCO_3_) to 1.5 mL/min. to promote the HCOO^–^ concentration at the cathodic liquid stream. This optimization established a balance between membrane hydration and K^+^ availability near the catalyst surface, enabling the collection of concentrated HCOO^–^ directly from the cathodic stream. We obtained an average HCOO^–^ FE of 82% (or 4.49 mmol/h) at 60 mA cm^−2^ during 100 h of CO_2_R with an average HCOO^–^ concentration of 3.4 molar (15 wt%) (Supplementary Fig. 55). This represents a 30-fold improvement in HCOO^–^ concentration with 100-hour stability along with a 2-fold increase in current density compared to the literature benchmarks reported in duration tests^6,27,41,42^ (Fig. 5e).

## Discussion

We present redox-modulated, stable, and active Sn-Bi/SnO_2_ surfaces on uniformly alloyed Bi_0.1_Sn crystals, and these catalysts show a combination of high activity and performance stability in the CO_2_-to-HCOO^–^ reduction exceeding 2400 h (100 days) of continuous operation. We use DFT calculations to explain that the stabilized active sites improve *OCHO binding energy and fine-tune *COOH and *H binding energy for selective HCOO^–^ production. We show stable production of concentrated HCOO^–^ (formate) of 3.4 molar with Bi_0.1_Sn catalysts in a solid-state CEM-based MEA system over 100 h. Our demonstration of a stable catalyst and system is a crucial step to deliver reliable and long-lasting CO_2_R technology. Further efforts will be necessary to increase CO_2_ single-pass yield and to the broader applicability of this system in the production of other C_2 + _liquid fuels and beyond.

## Methods

### Synthesis

Bi_*x*_Sn (*x* = 0.1, 0.2, 0.3), Bi, and Sn electrocatalysts were synthesized using thermal evaporation (SKY-RH400). In brief, to fabricate the Bi_*x*_Sn (*x* = 0.1, 0.2, 0.3) catalysts, different amounts of Sn and Bi were co-evaporated onto the PTFE substrates under the pressure of 10^−5^ Torr. The Bi evaporation rate was set to 0.1 nm s^−1^, and the Sn evaporation rate was set to 1 nm s^−1^, 0.5 nm s^−1^ and 0.3 nm s^−1^ to make the Bi_*x*_Sn (*x* = 0.1, 0.2, and 0.3) samples. The thickness of the deposited Bi_*x*_Sn layers was ~700 nm. The pure Bi and Sn films with the same film thicknesses were prepared at an evaporation rate of ~0.3 nm s^−1^ under the pressure of 10^−5^ Torr.

### Characterization

SEM images were taken using a Gemini500 SEM at an accelerating voltage of 2 kV. High-resolution transmission electron microscopy (HRTEM) and transmission electron microscopy-energy dispersive X-ray spectroscopy (TEM-EDX), SAED, and bright-field and dark-field TEM analyses were performed in a TEM (Tecn F20) with an accelerating voltage of 200 kV. X-ray photoelectron spectroscopy (XPS) studies were performed using PHI5000 VersaProbe. The binding energy data were calibrated relative to the C 1s signal at 284.6 eV. In the XPS depth profile studies, the etched depth is ~3–5 nm in each round of the soft Ar etching. X-ray powder diffraction (XRD) was carried out with a Bruker D8 Advance X-ray diffractometer using Cu Kα radiation at a scanning rate of 9°/min in the 2*θ* range from 20° to 80°.

### Electrochemical experiments

The CO_2_R experiments were performed in the KHCO_3_ and KOH electrolytes in a flow-cell device with a three-electrode setup. Different volumes of 10 M KOH were added to 1 M KHCO_3_ solution to adjust the pH to 11, 12, 13, and 14, respectively, confirming with a pH meter. An Ag/AgCl (in saturated KCl) electrode was used as a reference electrode; a nickel foam was used as a counter-electrode; Sn, Bi, and Bi_*x*_Sn electrodes were used as working electrodes. The three electrodes were connected to an electrochemical workstation (Metrohm Autolab). The flow rate of CO_2_ was set to 20–50 mL min^−1^. under standard conditions at the outlet end of the flow cell for all the experiments. The gaseous products were quantified using gas chromatography (GC, PerkinElmer) with a thermal conductivity detector (TCD) and a flame ionization detector (FID). The liquid products were detected using nuclear magnetic resonance (NMR, Bruker 400 M) with water peak suppression. To determine the Faraday efficiency of the liquid products, we quantified the liquid products in both analytes and catholyte by NMR. The electrolytes (on both sides) are also replaced at regular time intervals before the NMR tests. The CO_2_R performance of Bi, Sn, and Bi_0.1_Sn catalysts was also evaluated in MEA electrolysers. A commercial CO_2_R MEA electrolyser (Dioxide Materials) was used to accommodate the electrochemical reactions. The MEA electrolyser was composed of anode and cathode flow field plates with a serpentine-configuration flow field of 5 cm^2^ for the continuous supply of anolyte (0.1 M KHCO_3_) and humidified CO_2_ to each respective electrode. We used Bi/PTFE, Sn/PTFE, and Bi_0.1_Sn/PTFE as the cathode, an iridium oxide deposited titanium foam as the anode, and a solid-state cation-exchange membrane (CEM) for HCOO^–^ placed in between the cathode and anode. Before the electrochemical testing, the cathode electrodes (Bi, Sn, or Bi_0.1_Sn on PTFE) were taped to the stainless-steel flow field plate by using a copper frame for homogeneously distributing the electrical current. The anode (IrO_x_ on Ti foam) and cathode were physically separated by CEM (NafionTM 117, Fuel Cell Store) in the solid-state CEM-based MEA experiments and by AEM (Sustainion X37-50 membrane) in the AEM-based MEA experiments. Electrolyser bolts were tightened by applying an equal compression torque. Before experiments, the AEM was activated in 1 M KOH for more than 24 h. Upon completion of the electrolyser assembly, the anolyte (0.1 M KHCO_3_) flowed through the anode with a constant flow rate of 15 mL min^−1^, while the humidified CO_2_ was supplied from the gas diffusion electrode (GDL) back with a constant flow rate of 60 standard cubic centimetres per minute (sccm). The full-cell potentials are presented without IR correction. The gas samples were examined using GC (PerkinElmer Clarus 680) to calculate the FE of gas products. The liquid product, formate (when collected from the anodic stream from the AEM-based MEA) or HCOO^–^ (when collected from the cathodic stream from the solid-state CEM-based MEA), was collected from the anodic and cathodic streams simultaneously and analysed by an NMR spectroscopy (Agilent DD2 600 MHz) by using dimethylsulfoxide (DMSO) as the internal standard. The FE towards formate or formic acid at each current density was calculated by adding up both anodic and cathodic FEs. More details are discussed in the supplementary information.

### Density functional theory (DFT) calculation

The facets Bi (003), Sn (200), and Sn (101) are primarily exposed in the Bi, Sn, and Bi_0.1_Sn catalysts (Fig. 1); these facets were thus used to build the DFT models (Supplementary Fig. 10, Supplementary Table 1). We incorporated 1–8 Bi into 64 Sn crystals, as more than 8 Bi would de-stabilize the Sn crystal structure (Supplementary Figs. 12–15). All DFT calculations were performed with the Vienna ab initio simulation package (VASP)^43^. The DFT calculation details are included in the Supplementary Information.

## Supplementary information

Author Meta Data

Supplementary Information

## Data Availability

Source data to generate figures and tables are available from the corresponding authors.
