# Supplementary material for: Stable, active CO2 reduction to formate via redox-modulated stabilization of active sites
Source: Nat Commun. 2021 Sep 1;12:5223. doi: 10.1038/s41467-021-25573-9 (PMC8410779; doi:10.1038/s41467-021-25573-9)
Supplement: Supplementary file 2 — Supplementary Information [file 41467_2021_25573_MOESM2_ESM.pdf]

Supplementary Information

Stable, active CO_2 reduction to formate via redox-modulated stabilization of active sites

Le Li[1,†], Adnan Ozden[2,†], Shuyi Guo[3], F. Pelayo García de Arquer[4], Chuanhao Wang[1], Mingzhe Zhang[1], Jin Zhang[1], Haoyang Jiang[1], Wei Wang[3], Hao Dong[*,3], David Sinton[2], Edward H Sargent[*,4], Miao Zhong[*,1]

[1]College of Engineering and Applied Sciences, National Laboratory of Solid State Microstructures, Collaborative Innovation Center of Advanced Microstructure, Jiangsu Key Laboratory of Artificial Functional Materials, Nanjing University, Nanjing 210093, China.

[2]Department of Mechanical and Industrial Engineering, University of Toronto, Toronto, Ontario M5S 3G8, Canada

[3]Kuang Yaming Honors School & Institute for Brain Sciences, Nanjing University, Nanjing 210093, China.

[4]Department of Electrical and Computer Engineering, University of Toronto, 10 King's College Road, Toronto, ON, Canada, M5S 3G4.

[†]These authors contributed equally: Le Li, Adnan Ozden.

[(*)] Correspondence and requests for materials should be addressed to Miao Zhong (miaozhong@nju.edu.cn), Edward H. Sargent (ted.sargent@utoronto.ca) and Hao Dong (donghao@nju.edu.cn)

Supplementary Methods details.

Synthesis. Bi_xSn (x = 0.1, 0.2, 0.3), Bi, and Sn electrocatalysts were synthesized using thermal evaporation (SKY-RH400). To fabricate the Bi_xSn (x = 0.1, 0.2, 0.3) catalysts, different amounts of Sn and Bi were co-evaporated onto the PTFE substrates. A quartz crystal monitor was used to observe the thicknesses of Bi_xSn films during the evaporation. In our experiments, 1.16 g Bi powder and 0.95 g Sn powder were placed in a molybdenum boat inside the deposition chamber. Metal powders were slowly melted under a pressure of 10^{-5} Torr. The Bi evaporation rate was set to 0.1 nm s^{-1}, and the Sn evaporation rate was set to 1 nm s^{-1}, 0.5 nm s^{-1} and 0.3 nm s^{-1} to make the Bi_xSn (x = 0.1, 0.2, and 0.3) samples. The thickness of the deposited Bi_xSn layers was ~700 nm. The pristine Bi and Sn films with the same film thicknesses were prepared at an evaporation rate of ~ 0.3 nm s^{-1} under the pressure of 10^{-5} Torr.

Characterization. SEM images were taken using a Gemini500 SEM at an accelerating voltage of 2 kV. High-resolution transmission electron microscopy (HRTEM) and transmission electron microscopy-energy dispersive X-ray spectroscopy (TEM-EDX), selected area electron diffraction (SAED), and bright-field and dark-field TEM analyses were performed in a TEM (Tecn F20) with an accelerating voltage of 200 kV. X-ray photoelectron spectroscopy (XPS) studies were performed using PHI5000 VersaProbe. XPS depth profile studies were etched using energy of 2000 eV, and the etch time was 120s each time. The binding energy data were calibrated relative to the C 1s signal at 284.6 eV. X-ray powder diffraction (XRD) was carried out with a Bruker D8 Advance X-ray diffractometer using Cu Kα radiation at a scanning rate of 9°/min in the 2θ range from 20° to 80°.

Electrochemical experiments. The CO_2R experiments were performed in the $KHCO_3$ and KOH electrolytes in a flow-cell device with a three-electrode setup. Different volumes of 10 M KOH were added to 1 M $KHCO_3$ solution to adjust the pH to 11, 12, 13, and 14, respectively, confirming with a pH meter. An Ag/AgCl (in saturated KCl) electrode was used as a reference electrode; a nickel foam was used as a counter-

electrode; Sn, Bi, and Bi_xSn electrodes were used as working electrodes. The three electrodes were connected to an electrochemical workstation (Metrohm Autolab). The flow rate of CO_2 was set to 20–50 mL min^{-1} under standard conditions at the outlet end of the flow cell for all the experiments. The linear sweep voltammetry (LSV) studies were carried out at a sweep rate of 10 mV s^{-1} in the potential range of 0 to –2 V_{RHE}. The electrochemically active surface area (ECSA) measurements were conducted using cyclic voltammetry (CV) scans at different rates from 40 to 200 mV s^{-1} with a 40 mV s^{-1} increment speed from –0.077 V_{RHE} to –0.177 V_{RHE}; within this potential range, the Faradic process is excluded. Electrochemical impedance spectroscopy (EIS) was carried out to estimate the electrolyte resistance for IR compensation. The electrolyte resistance was measured at open circuit potential in a frequency range from 10 MHz to 0.1 Hz with an amplitude of 10 mV. All of the electrode potentials vs. the Ag/AgCl electrode were converted to the potentials vs. reversible hydrogen electrode (RHE) using the following equation (1):

$$E_{RHE} = E_{Ag/AgCl} + 0.197\ V + 0.059 \times pH\ , \qquad (1)$$

The ESCA is calculated by the following equation (2):

$$A_{ECSA} = \frac{\text{Specific capacitance}}{40\ \mu Fcm^{-2}\ cm_{ECSA}{}^{-2}}, \qquad (2)$$

The specific capacitance of the sample was obtained by CV. It was carried out at different scan rates in the range of –0.077 V_{RHE} to –0.177 V_{RHE}. 40 mF cm^{-2} is the specific capacitance of a flat surface for metallic and semiconducting materials with 1 cm^2 of the real surface area in the aqueous electrolyte[1].

The gaseous products were quantified using gas chromatography (GC, PerkinElmer) with a thermal conductivity detector (TCD) and a flame ionization detector (FID). The liquid products were detected using nuclear magnetic resonance (NMR, Bruker 400 M) with water peak suppression. Typically, 1 mL of the electrolyte after electrolysis was mixed with 200 μL of D_2O (Acros Organics1, 99.8 at.% D) and 100 μL 1/1000 dimethyl sulfoxide (Aladdin, 99.9%), used as internal standard. An aliquot of 1 mL of this solution served as the sample used for NMR.

The Faradaic efficiency (FE) of the liquid product ($HCOO^-$) were calculated using

equation (3):

$$\mathrm{FE}_{HCOO^-} = \frac{\mathrm{n} * \mathrm{F} * \mathrm{V} * \mathrm{c}}{1000 * \mathrm{M} * \mathrm{Q}}, \qquad (3)$$

where n is the transfer electron number, F is the Faraday efficiency constant (96485 C mol^{-1}), c is the mass concentration of the acid root generated by the reaction (in mg L^{-1}), V is the electrolyte solution volume (in L), M is the molar mass of formic acid (46.03 g mol^{-1}), and Q is the total amount of charge consumed by the entire reaction as monitored by the electrochemical workstation (in coulombs). The FEs of the gaseous products were calculated using equation (4):

$$\mathrm{FE}_{gas} = \frac{\mathrm{n} * \mathrm{F} * \mathrm{V}}{1000 * 22.4 * \mathrm{Q}}, \qquad (4)$$

where n is the transfer electron number, F is the Faraday efficiency constant (96485 C mol^{-1}), V is the generated volume of gaseous products (in L), and Q is the total amount of charge consumed by the entire reaction as monitored by the electrochemical workstation (in coulombs).

All measurements were conducted at room temperature under ambient pressure. The half-cell energy conversion efficiency (CEE, also called cathodic energy efficiency) is calculated using equation (5):

$$\mathrm{CEE}_{formate} = \frac{(1.23 - \mathrm{E_{formate}}) * \mathrm{FE_{formate}}}{1.23 - \mathrm{E_{cathode}}}, \qquad (5)$$

where $E_{formate}$ of –0.199 V_{RHE} is the standard potential of the formate formation. $FE_{formate}$ is the measured formate Faradaic efficiency. $E_{cathode}$ is the applied potential vs. RHE.

To determine the Faraday efficiency of the liquid products, we quantified the liquid products in both anolyte and catholyte by NMR. The electrolytes (on both sides) are changed at regular time intervals before the NMR tests.

Density functional theory (DFT) calculation. The facets Bi (003), Sn (200), and Sn (101) are primarily exposed in the Bi, Sn and $Bi_{0.1}Sn$ catalysts (Figure 1); these facets were thus used to build the DFT models (Supplementary Fig. 10, Supplementary Table 1). We incorporated 1–8 Bi into 64 Sn crystals, as more than 8 Bi would de-stabilize the Sn crystal structure (Supplementary Figs. 12–15). This agrees with the EDX results that Bi precipitates in the $Bi_{0.2}Sn$ and $Bi_{0.3}Sn$ samples.

The unit cell parameters of the Sn crystal[2] are a = b = 5.831, c = 3.182. Starting from this unit cell, a 2 × 4 × 4 supercell was constructed, with an additional vacuum space of 20 Å added along the c direction to build a slab model with a total of 64 atoms.

All DFT calculations were performed with the Vienna ab initio simulation package (VASP)[3]. The projector-augmented wave[4, 5] was used to describe the ion-electron interaction in the periodic boundary condition, and the generalized gradient approximation and Perdew−Burke−Ernzerhof[6] was used. After benchmark calculations, the cutoff energy was set to be 460 eV, and the k-points were set at 3 × 2 × 1, and SIGMA was set to be 0.2 eV. The convergence criterion for the energy difference between two steps was 10^{-4} eV. Spin correction was not used. During the structural optimization, the top two layers of atoms in the system were relaxed and the remaining two layers in the bulk phase were fixed. The open-source code vaspkit[7] was used for state of density and the thermodynamics (298 K and 1 atm) calculations. For Gibbs free energy, the following equation was used:

$$G = H - TS = E_{DFT} + E_{ZPE} + \int_{O}^{298} C_v dT - TS \quad , \quad (6)$$

where E_{DFT} is the electronic energy and E_{ZPE} is the zero-point vibrational energy. The third and the fourth terms are heat capacity and correction to entropy, respectively, obtained from frequency calculations. For gas molecules, these data were adopted from earlier work from Klinkova *et al*[8]. The following corrections were applied to partially counterbalance the overestimation from DFT calculations[9, 10]: CO_2 (0.45 eV), HCOOH (0.20 eV), H_2 (-0.09 eV), and the adsorbed COOH* (0.20 eV). The solvation was calculated with the vassal[11], a method of calculating implicit models in a self-consistent continuous model that incorporates solvation into a software package in VASP.

The binding energy E_{ad*} was estimated by using the following equations:

$$E_{COOH*} = E_{COOH*} - E_{surf} - (E_{HCOOH} - 1/2E_{H_2}) \quad (7)$$

$$E_{HCOO*} = E_{HCOO*} - E_{surf} - (E_{HCOOH} - 1/2E_{H_2}) \quad (8)$$

E_{surf} and $E_{molecule}$ represent the energy of the bound state and the isolated molecule, respectively.

We take the following reaction (9) as an example to show the calculation of free

energy changes by using equation (10), where e is the charge number, and U is the applied voltage, for the calculation of free energy of H^+ and e^-, the computational hydrogen electrode (CHE) model[12] was used to calculate the free energy of proton and electron:

$$HCOO* + H^+ + e^- \leftrightarrow HCOOH + * \quad (9)$$

$$\Delta G = G_* + G_{HCOOH} - G_{HCOO*} - (G_{H^+} + G_{e^-}) = G_* + G_{HCOOH} - G_{HCOO*} - \left(\frac{1}{2}G_{H_2} - eU\right) \quad (10)$$

CO_2R performance in the flow-cell electrolyser. To verify the CO_2R stability of the $Bi_{0.1}Sn$ catalyst, carbon nanoparticles (NPs) and graphite were sprayed on top of the $Bi_{0.1}Sn$/PTFE electrode to form a hybrid graphite/carbon NPs/$Bi_{0.1}Sn$/PTFE structure. This coating of carbon nanoparticles and graphite helps to better distribute the electrical current and electrolyte ions over the catalyst surfaces during the reaction. We systematically studied the CO_2R performance of NPs/$Bi_{0.1}Sn$/PTFE in $KHCO_3$ and KOH electrolytes at pH 11. The Bi catalysts were studied under the same conditions. The surfaces of Bi and $Bi_{0.1}Sn$ showed completely different changes after the same reaction time of 70 h. Bi reconstructed to small nanoparticles. The small nanoparticles eventually all peeled off from the substrates after 100 hours of reaction. In contrast, $Bi_{0.1}Sn$ remained unchanged during the long-term stability test.

CO_2R performance in MEA electrolysers. The CO_2R performance of Bi, Sn, and $Bi_{0.1}Sn$ catalysts was evaluated in MEA electrolysers. A potentiostat with a current booster (Metrohm Autolab, 10 A) was used to apply the current and voltage, a commercial CO_2R MEA electrolyser (Dioxide Materials) was used to accommodate the electrochemical reactions, a mass flow controller was used to set the flow rate of CO_2, a humidifier was used to supply the humidified CO_2, and a peristaltic pump with silicone tubing was used to circulate the anolyte. The MEA electrolyser was composed of anode and cathode flow field plates with a serpentine-configuration flow field of 5 cm^2 for the continuous supply of anolyte (0.1 M $KHCO_3$) and humidified CO_2 to each

respective electrode. We used Bi/PTFE, Sn/PTFE, and $Bi_{0.1}Sn$/PTFE as the cathode, an iridium oxide deposited titanium foam as the anode, and a solid-state cation-exchange membrane (CEM) for $HCOO^-$ placed in between the cathode and anode. Before the electrochemical testing, the cathode electrodes (Bi, Sn, or $Bi_{0.1}Sn$ on PTFE) were taped to the stainless-steel flow field plate by using a copper frame for homogeneously distributing the electrical current. The anode (IrO_x on Ti foam) and cathode were physically separated by CEM (NafionTM 117, Fuel Cell Store) in the solid-state CEM-based MEA experiments and by AEM (Sustainion X37-50 membrane) in the AEM-based MEA experiments. Electrolyser bolts were tightened by applying an equal compression torque. Before experiments, the AEM was activated in 1 M KOH for more than 24 hours; the CEM was activated by following a procedure described in ref. [13] and stored in deionized water. Upon completion of the electrolyser assembly, the anolyte (0.1 M $KHCO_3$) flowed through the anode with a constant flow rate of 15 mL min^{-1} using a peristaltic pump, while the humidified CO_2 was supplied from the gas diffusion electrode (GDL) back with a constant flow rate of 60 standard cubic centimetres per minute (sccm). We measured the CO_2 flow rate at the outlet end of the MEA system. The CO_2R was then initiated by applying a constant current density (–30, –60, –90, –120, –150 and –180 mA cm^{-2}). The corresponding cell potentials for the current densities of interest were recorded with continuous monitoring of the gas and liquid products. The full-cell potentials are presented without IR correction. The gas products were collected from the cathodic gas stream via a gas-tight syringe (Hamilton chromatography syringe) in a constant volume of 1 mL. The gas samples were examined using GC (PerkinElmer Clarus 680) to calculate the FE of gas products, including hydrogen, carbon monoxide, methane, and ethylene. For each current density tested, the gas products were collected upon complete stabilization of the cell voltage at least three times. The liquid product, formate (when collected from the anodic stream from the AEM-based MEA) or $HCOO^-$ (when collected from the cathodic stream from the solid-state CEM-based MEA), was collected from the anodic and cathodic streams simultaneously and analysed by a NMR spectroscopy (Agilent DD2 600 MHz) by using dimethylsulfoxide (DMSO) as the internal standard. The FE towards formate or formic

acid at each current density was calculated by adding up both anodic and cathodic FEs.

Supplementary Figures

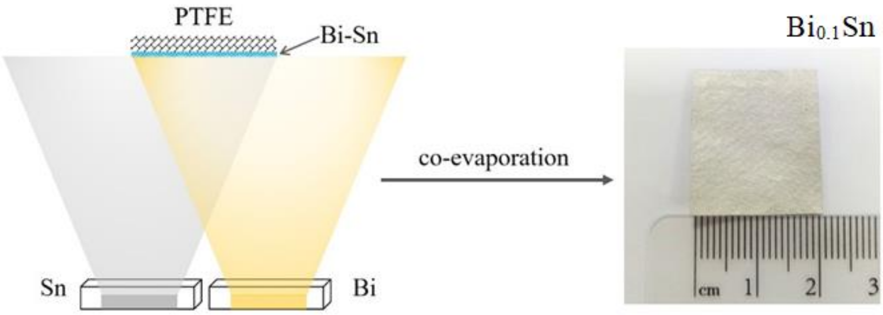

Supplementary Figure 1. Synthesis of Sn, Bi, and Bi_xSn (x = 0.1, 0.2, 0.3) catalysts on gas diffusion layers (PTFE substrates).

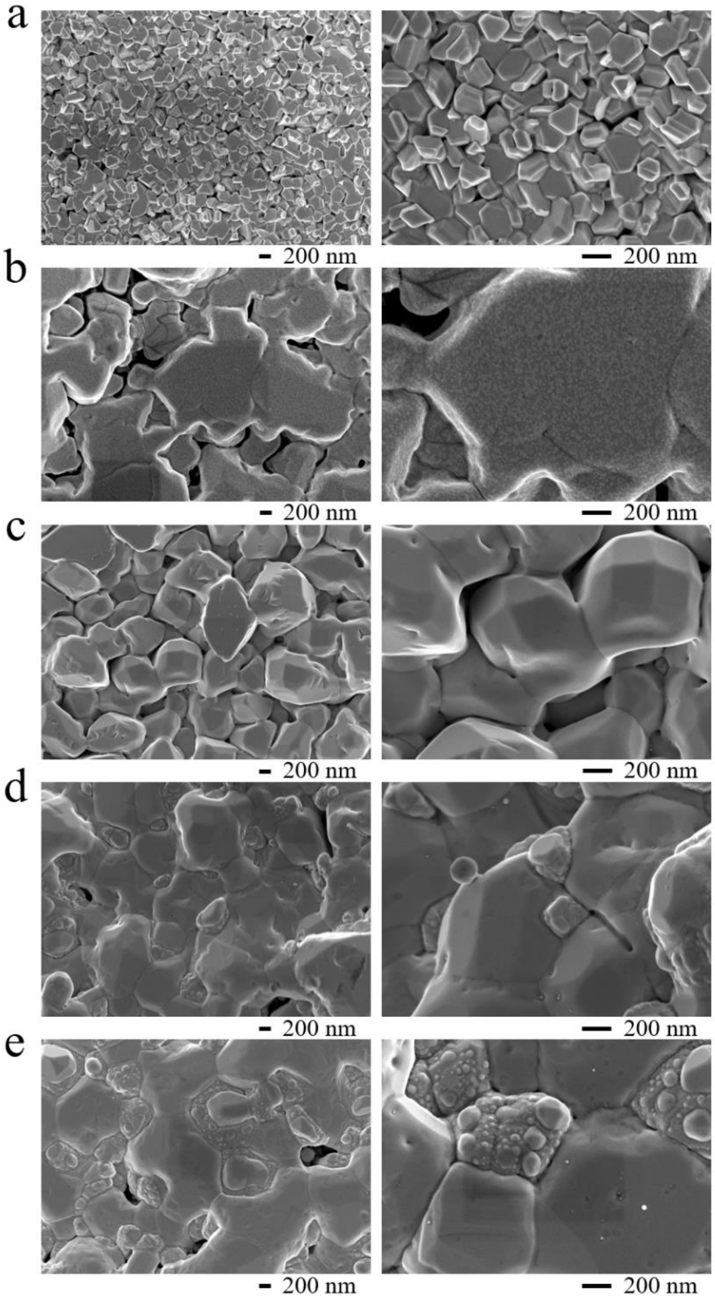

Supplementary Figure 2. SEM images of the prepared **a**, Bi. **b**, Sn. **c**, $Bi_{0.1}Sn$. **d**, $Bi_{0.2}Sn$ and **e**, $Bi_{0.3}Sn$ catalysts.

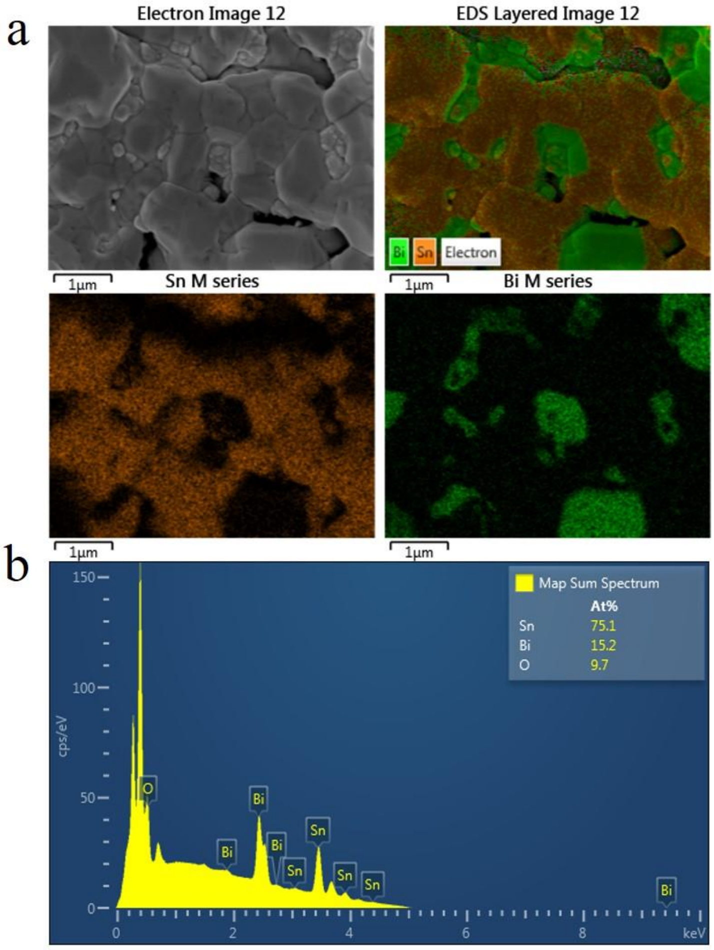

Supplementary Figure 3. EDX analysis in SEM of the $Bi_{0.2}Sn$ catalyst. **a**, EDX elemental mapping results. **b**, EDX spectrum with the estimated elemental concentrations.

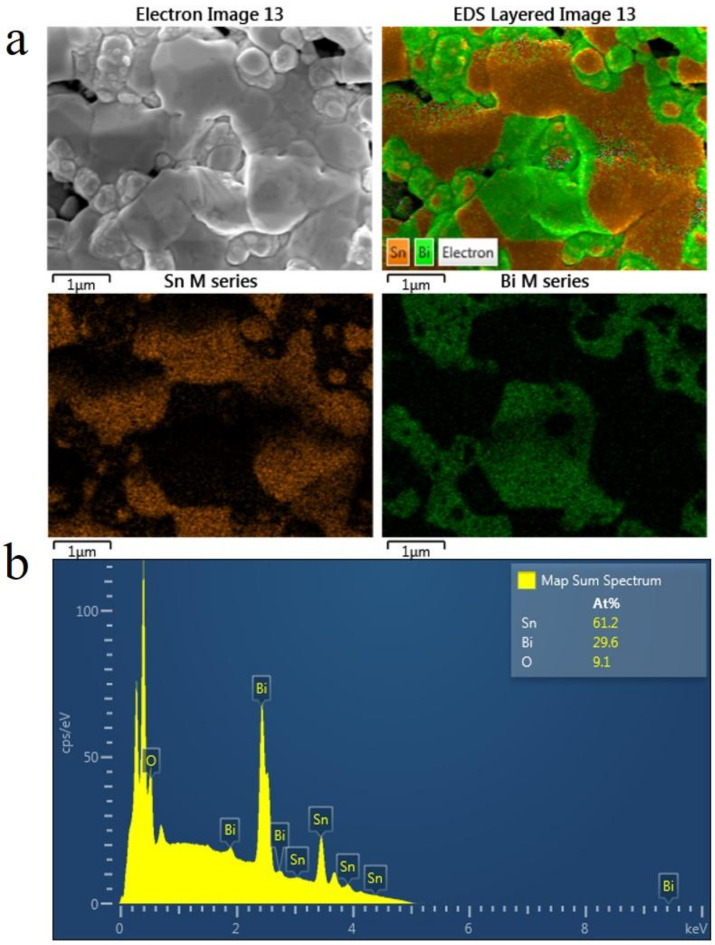

Supplementary Figure 4. EDX analysis in SEM of the $Bi_{0.3}Sn$ catalyst. **a**, EDX elemental mapping results. **b**, EDX spectrum with the estimated elemental concentrations.

a

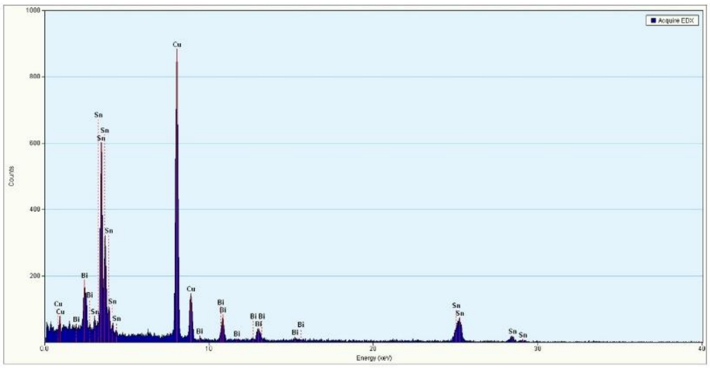

b

Element	Weight %	Atomic %	Uncert. %	Correction	k-Factor
Sn(K)	79.57	87.27	1.84	0.78	10.461
Bi(L)	20.42	12.72	0.48	0.99	4.797

Supplementary Figure 5. TEM-EDX analysis of the $Bi_{0.1}Sn$ catalyst. **a**, EDX elemental mapping results. **b**, EDX spectrum with the estimated elemental concentrations.

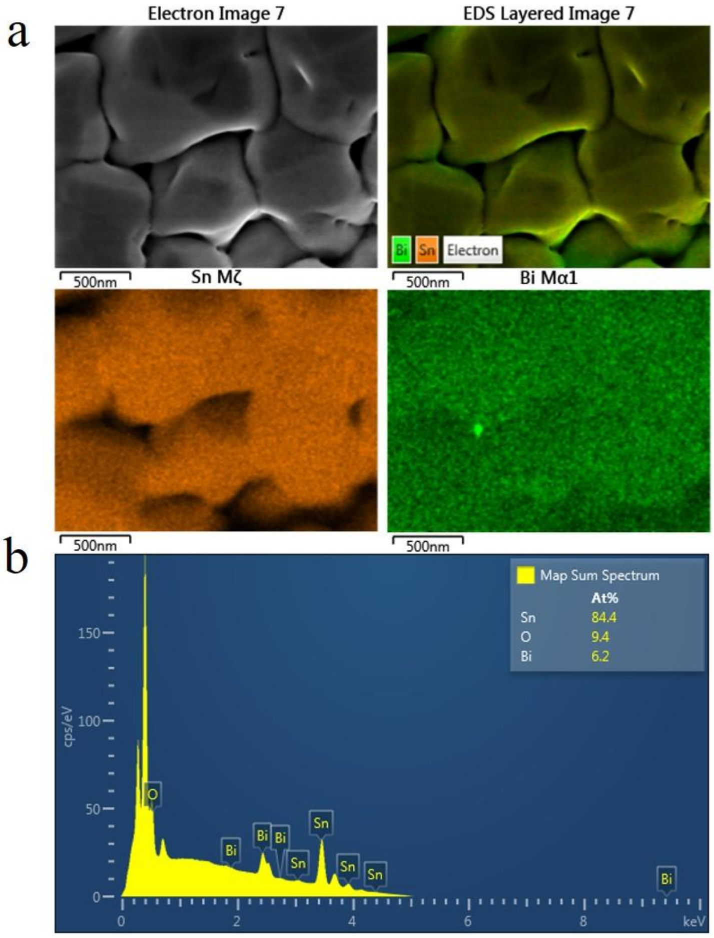

Supplementary Figure 6. EDX analysis of the $Bi_{0.1}Sn$ catalyst. **a**, EDX elemental mapping results. **b**, EDX spectrum with the estimated elemental concentrations. We average Bi:Sn ratios from multiple regions measured, and the result shows a Bi:Sn ratio close to 0.1. The oxygen signal may come from the PTFE substrate (as it contains oxygen in its chemical structure) and it may also come from our sample surfaces or the sample holder.

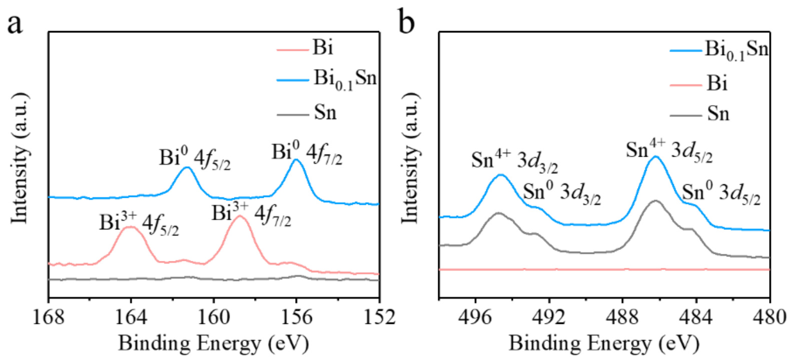

Supplementary Figure 7. XPS narrow scans of **a**, Bi 4f spectra and **b**, Sn 3d spectra of the Bi, Sn and $Bi_{0.1}Sn$ catalysts.

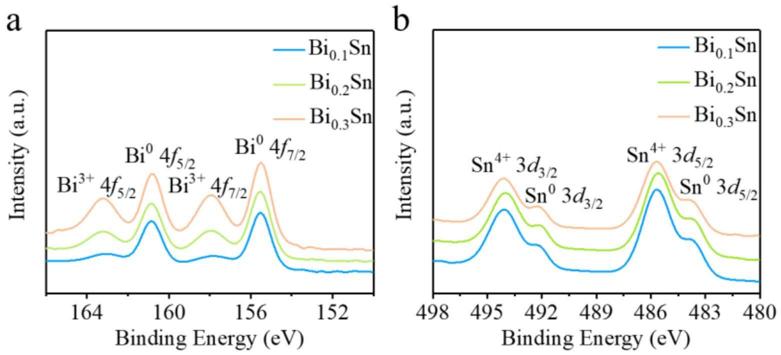

Supplementary Figure 8. XPS narrow scans of **a**, Bi 4f spectra and **b**, Sn 3d spectra of the $Bi_{0.1}Sn$, $Bi_{0.2}Sn$ and $Bi_{0.3}Sn$ catalysts.

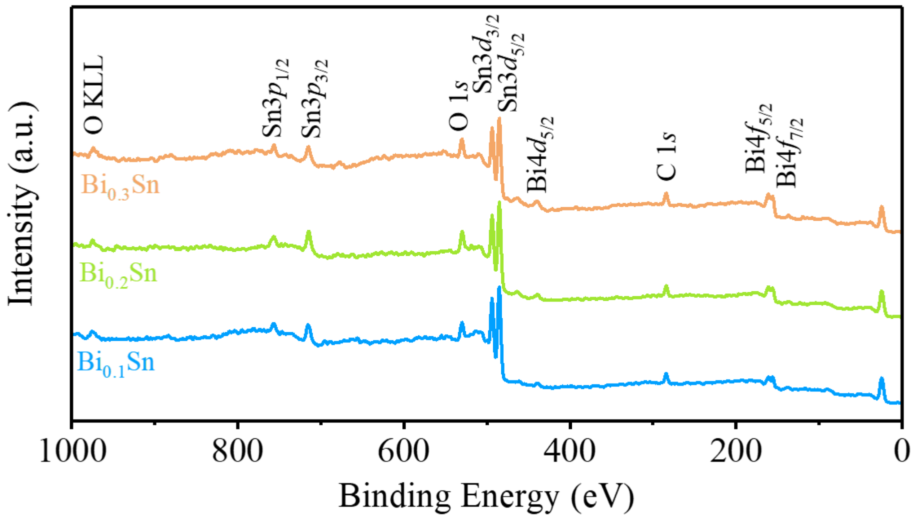

Supplementary Figure 9. XPS survey spectra of $Bi_{0.1}Sn$, $Bi_{0.2}Sn$ and $Bi_{0.3}Sn$.

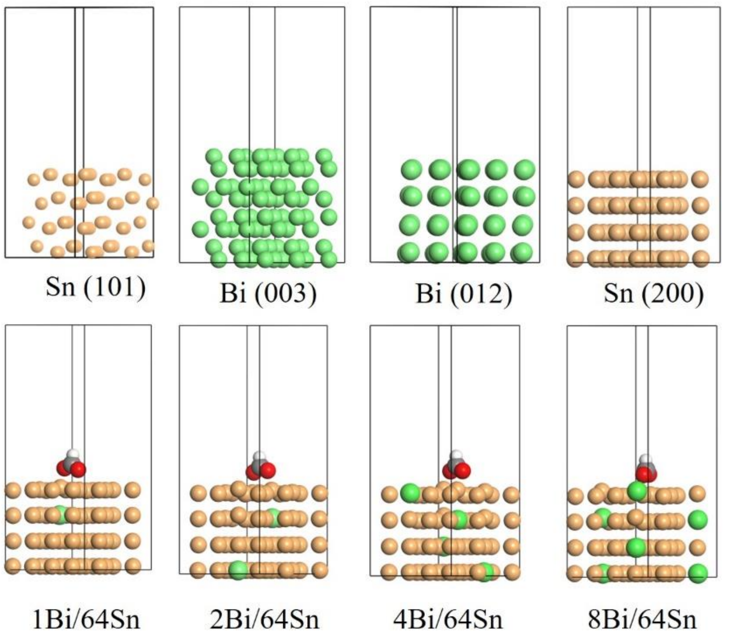

Supplementary Figure 10. The slab model used for calculations. Top panel: the Sn (101), Bi (003), Bi (012), and Sn (200) surface structures. Bottom panel: an *OCHO molecule adsorbs on the corresponding surface.

According to the SAED in TEM and XRD characterizations, Sn (200), Sn (101), Bi (003), and Bi (012) surfaces are used to build slab models for calculation. The calculated most active sites were the above Bi_4Sn_{64} slab with suitable *OCHO binding energy.

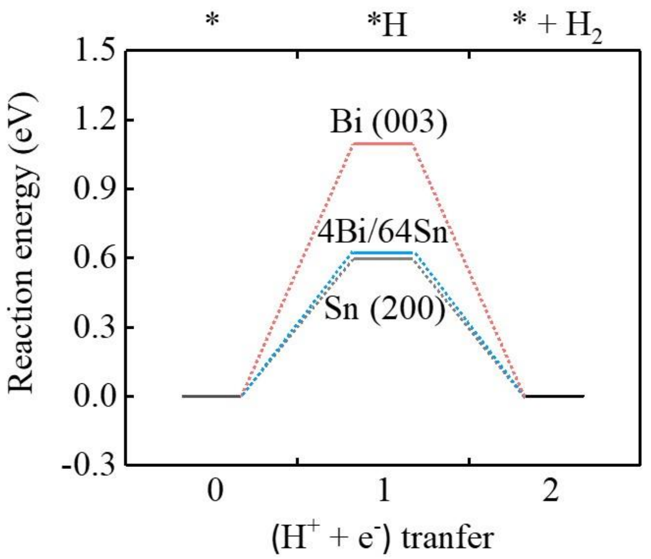

Supplementary Figure 11. Reaction energy profiles for H_2 formation on Bi (003), Sn (200), and Bi_4Sn_{64} (200) without applying any external potential (U = 0 eV).

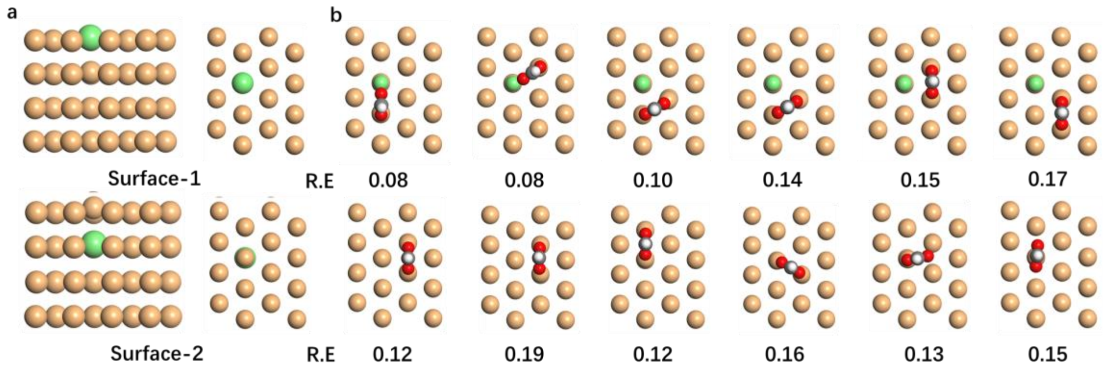

Supplementary Figure 12. DFT calculations of *OCHO on 1Bi/64Sn (200) surface. **a,** Side (left) and top (right) views of the slab model with 1 Bi at the first (top panel) or the second (bottom panel) layer of the Sn (200) surface in the absence of *OCHO. Bi, Sn, O, and H atoms are represented by green, brown, red, and white spheres, respectively, and the size of the Bi atoms is slightly enlarged for display. **b,** Different configurations and the corresponding reaction energies (in eV) of *OCHO bound to the 1Bi/64Sn (200) structures.

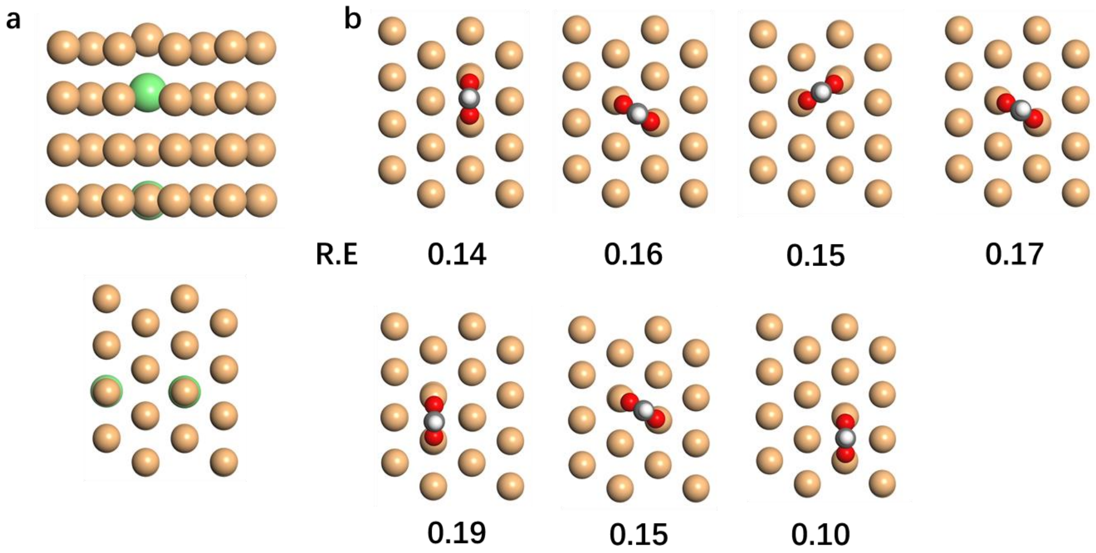

Supplementary Figure 13. DFT calculations of *OCHO on 2Bi/64Sn (200) surface. **a,** Side (top panel) and top (bottom panel) views of the slab model with 1 Bi atom at the second lay and another Bi atom at the fourth layers of the Sn (200) surface in the absence of *OCHO. Bi, Sn, O, and H atoms are represented by green, brown, red, and white spheres, respectively, and the size of the Bi atoms is slightly enlarged for display. **b,** Different configurations and the corresponding reaction energies (in eV) of *OCHO bound to the 2Bi/64Sn (200) facet.

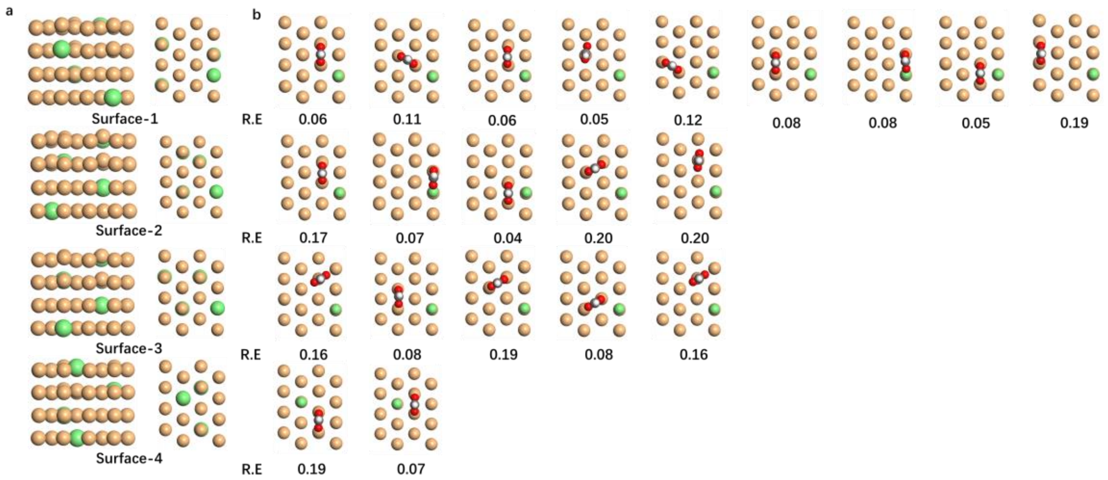

Supplementary Figure 14. DFT calculations of *OCHO on 4Bi/64Sn (200) surface. **a,** Side (left) and top (right) views of the slab model with 1 Bi atom at each layer of the Sn (200) surface in the absence of *OCHO. Bi, Sn, O, and H atoms are represented by green, brown, red, and white spheres, respectively, and the size of the Bi atoms is slightly enlarged for display. The four panels from top to bottom represent four different configurations of the 4Bi/64Sn (200) system. **b,** Different configurations and the corresponding reaction energies (in eV) of *OCHO bound to the 4Bi/64Sn (200) facet.

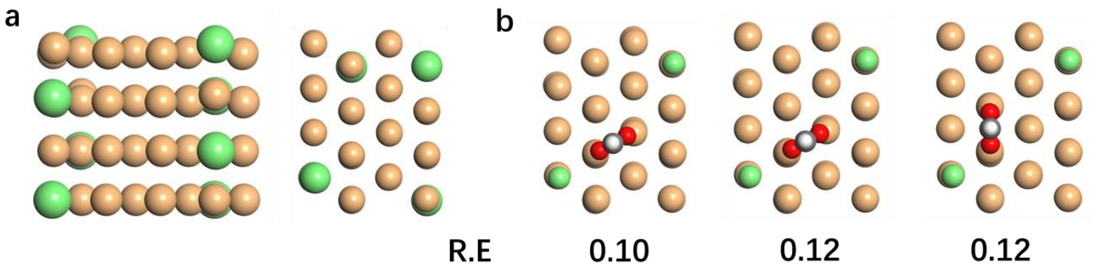

Supplementary Figure 15. DFT calculations of *OCHO on 8Bi/64Sn (200) surface. **a,** Side (left) and top (right) views of the slab model with 2 Bi atoms at each layer of the Sn (200) surface in the absence of *OCHO. Bi, Sn, O, and H atoms are represented by green, brown, red, and white spheres, respectively, and the size of the Bi atoms is slightly enlarged for display. **b,** Different configurations and the corresponding reaction energies (in eV) of *OCHO bound to the 8Bi/64Sn (200) facet.

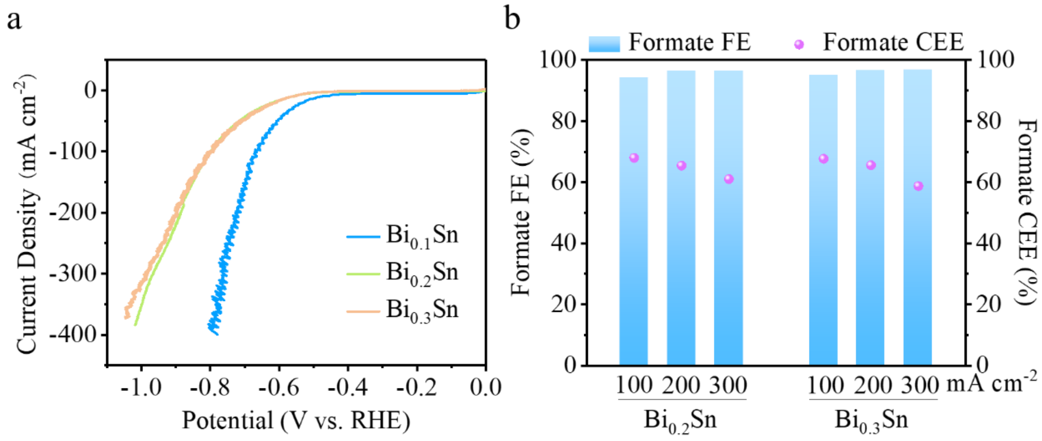

Supplementary Figure 16. **a,** Linear sweep voltammetry (LSV) curves of the Bi_xSn (x = 0.1, 0.2, 0.3) catalysts in 1 M KOH electrolyte. **b,** The corresponding formate FEs and CEEs under different current densities of $Bi_{0.2}Sn$ and $Bi_{0.3}Sn$.

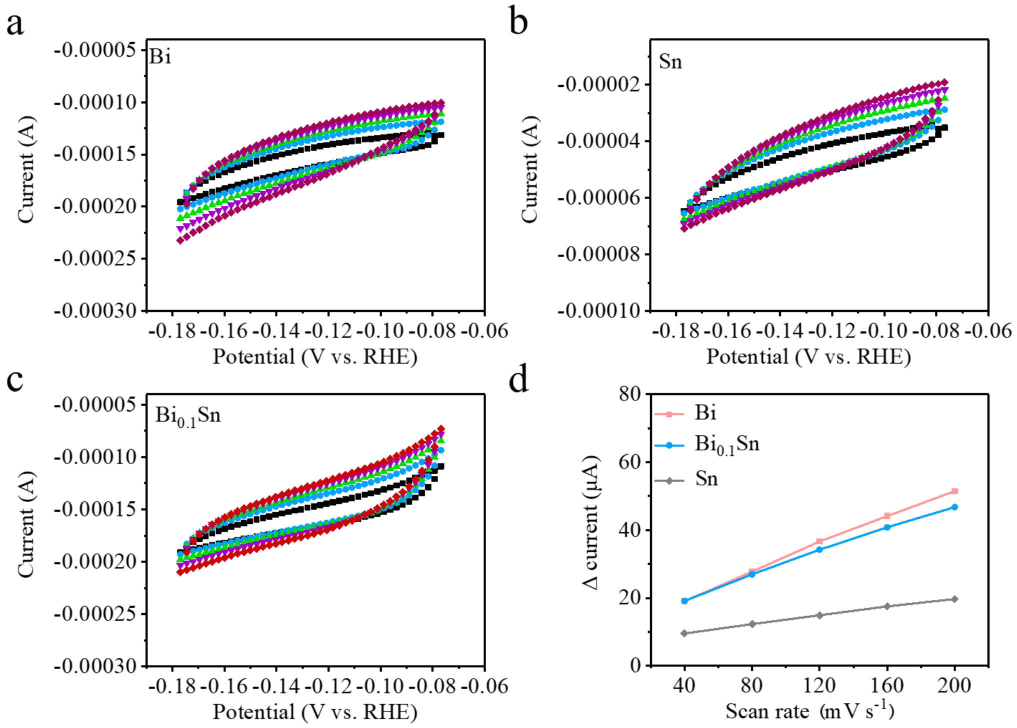

Supplementary Figure 17. CV curves of **a**, Bi. **b**, Sn and **c**, $Bi_{0.1}Sn$ catalysts. **d**, the variation of current as a function of CV scan rates for Bi, Sn and $Bi_{0.1}Sn$ catalysts with the same geometric area for each sample in the same electrolyte of 1 M KOH. The specific capacitances of Bi (406 uF cm^{-2}) and $Bi_{0.1}Sn$ (346 uF cm^{-2}) catalysts were larger than that of Sn catalysts (128 uF cm^{-2}), indicating that the ECSA of $Bi_{0.1}Sn$ is 2.7x larger than that of Sn.

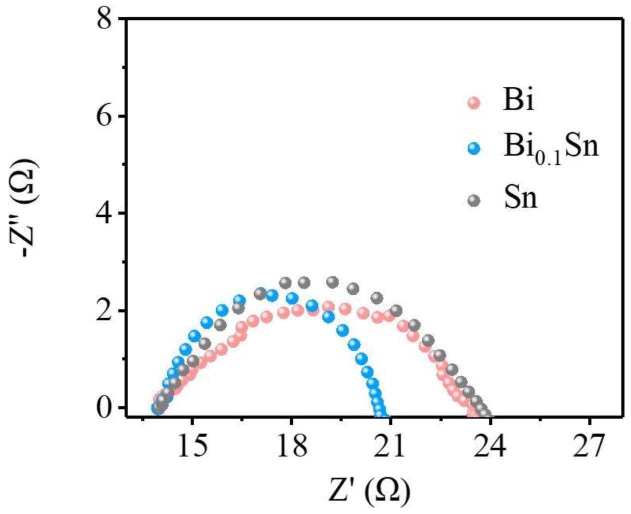

Supplementary Figure 18. Electrochemical impedance spectroscopy (EIS) results of Bi, Sn and $Bi_{0.1}Sn$ catalysts.

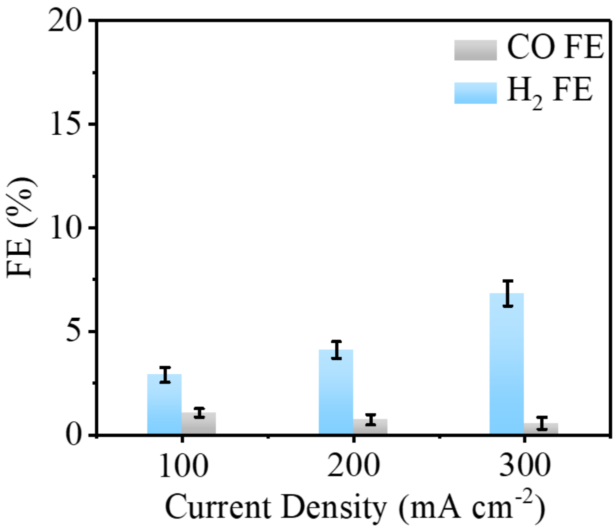

Supplementary Figure 19. H_2 and CO FEs of $Bi_{0.1}Sn$ catalyst in 1 M KOH electrolyte under different current densities. Error bars correspond to the standard deviation of five independent measurements.

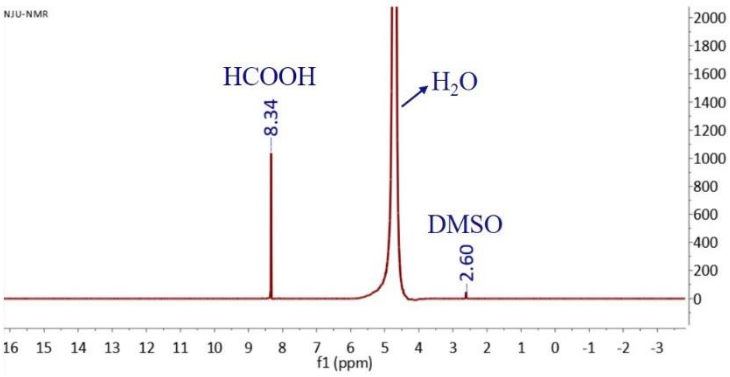

Supplementary Figure 20. The ^{1}H NMR spectrum of the reaction electrolyte after 120 hours CO_2R using a $Bi_{0.1}Sn$ electrode.

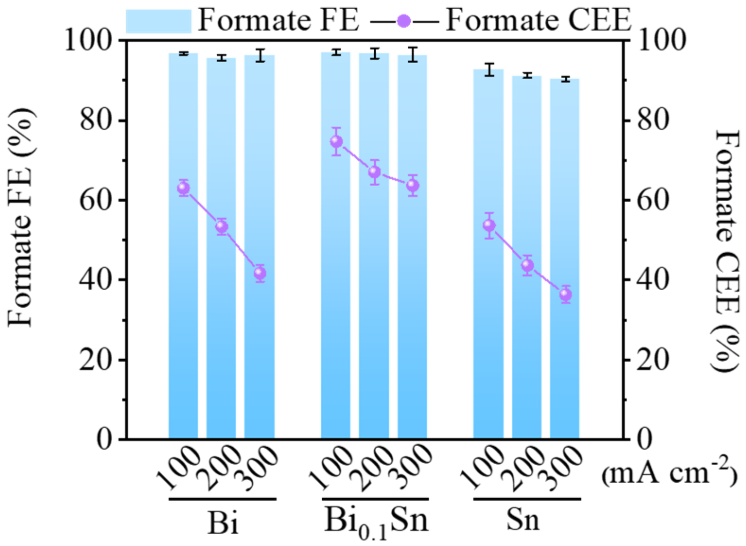

Supplementary Figure 21. The formate FEs and CEEs under different current densities of $Bi_{0.1}Sn$, Bi, and Sn in 1 M KOH electrolyte. Error bars correspond to the standard deviation of five independent measurements.

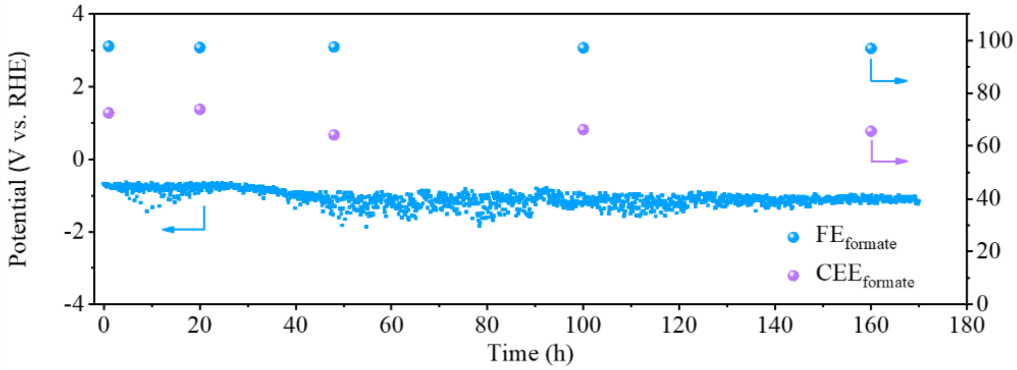

Supplementary Figure 22. The CO_2-reduction chronopotentiometry curve (blue line), formate FE (blue dots), and CEE (purple dots) of the graphite/carbon NPs/$Bi_{0.1}Sn$/PTFE catalyst in a 1 M KOH electrolyte at 100 mA cm^{-2}.

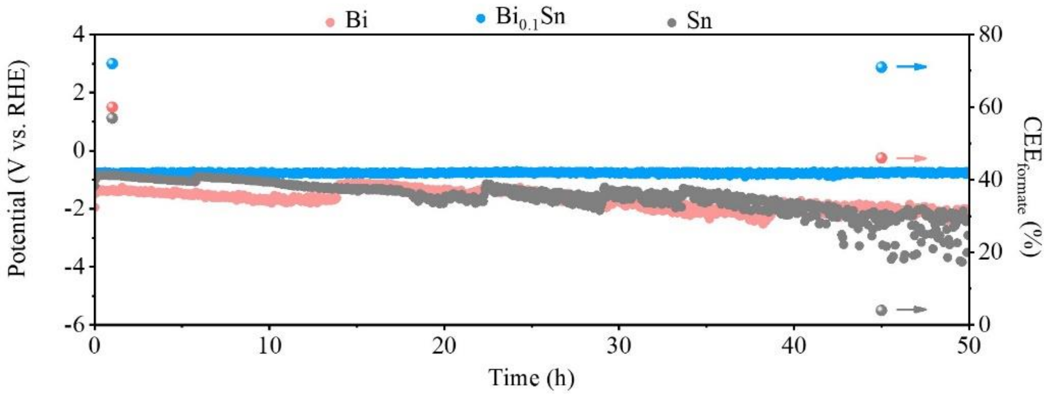

Supplementary Figure 23. The CO_2R performance of Bi, Sn and $Bi_{0.1}Sn$ catalysts with the same carbon NPs and graphite coatings in 1 M KOH at pH = 14 at an applied current density of 100 mA cm^{-2}.

As control experiments, we studied the CO_2R stability of Bi and Sn catalysts with the same carbon NPs and graphite coatings under the same electrochemical conditions. SEM images reveal that small Bi nanoparticles were formed all over the catalyst layer, likely due to the Bi surface reconstruction during CO_2R (Supplementary Fig. 24). The electrical resistance of Bi_2O_3/Bi nanoparticles was increased, and the CEE was decreased. Small Bi_2O_3/Bi nanoparticles partially peeled away from the PTFE, leaving cracks in the catalyst layer, which further increase electrical resistance.

Compared to Bi, Sn consistently produced in total ~20% H_2 and CO (Supplementary Fig. 25). It needed a large overpotential (Supplementary Fig. 23). The overall formate CEE is lower. Note that (i) the pH near the surfaces is high compared to that in the electrolyte bulk at a high current density during CO_2R, and (ii) catalyst geometry could cause the non-uniform distribution of OH^- ions near the surfaces. We found there was increased H_2 production with Sn catalysts during the 50-hour stability test at pH 14 (Supplementary Fig. 25).

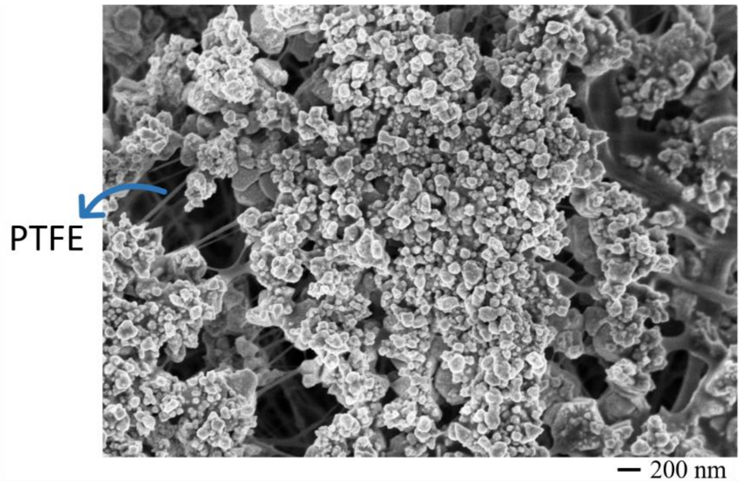

Supplementary Figure 24. The SEM images of Bi catalysts after 50 hours CO_2R in 1 M KOH electrolyte at pH 14 at an applied current density of 100 mA cm^{-2}.

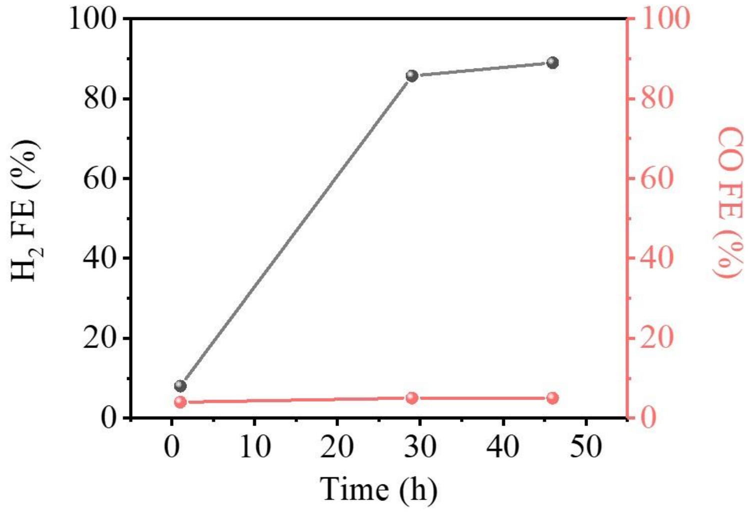

Supplementary Figure 25. FE of H_2 and CO for CO_2R using Sn catalysts in 1 M KOH electrolyte at pH 14 at an applied current density of 100 mA cm^{-2}.

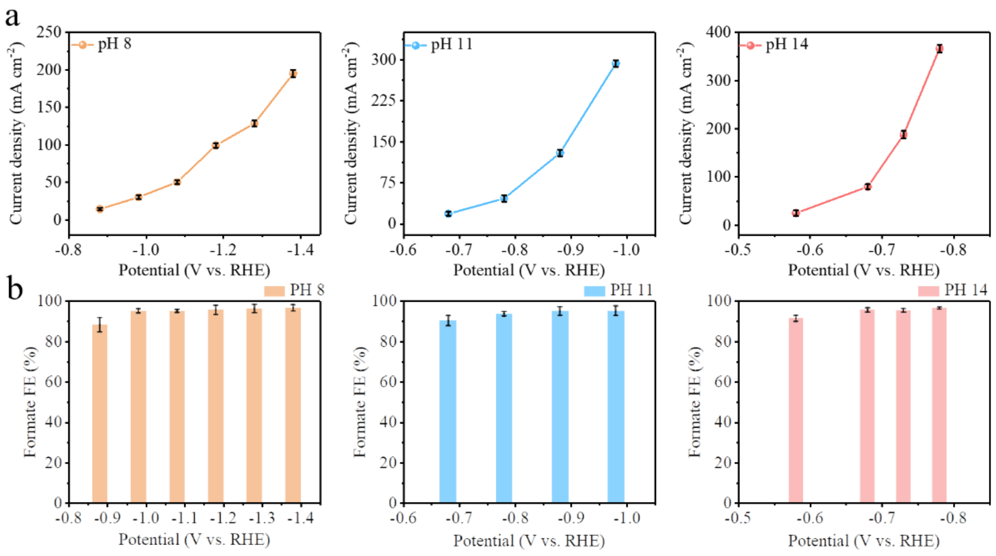

Supplementary Figure 26. Current density (a) and formate FEs (b) of $Bi_{0.1}Sn$ catalysts measured in 1 M $KHCO_3$ and KOH electrolytes at different pH (8, 11,14). Compared to the conventional “H-cell”, the major advantage of the flow cell is to achieve a large CO_2R current density. We therefore compare the formate performance at a relatively high current density. In addition, the counter-electrode reaction in the full CO_2R electrolysis is water oxidation, which is RHE dependent. We therefore use the RHE scale to unite the potential on both sides. Error bars correspond to the standard deviation of three independent measurements.

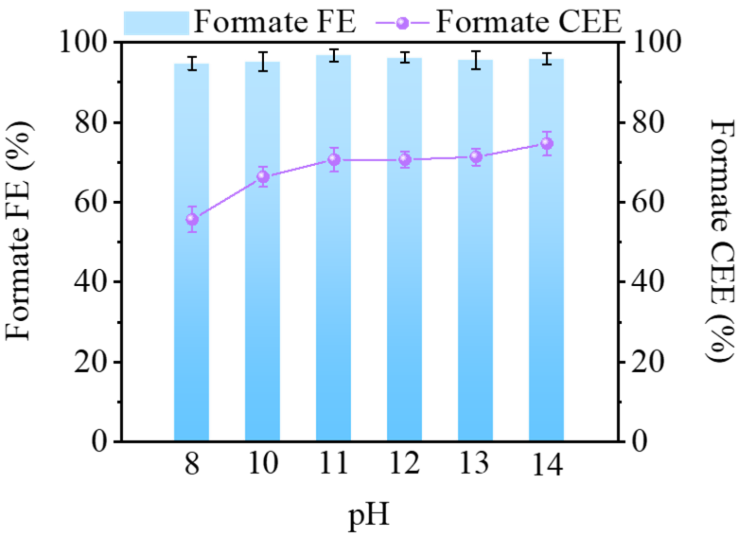

Supplementary Figure 27. The formate FEs and CEEs in 1 M $KHCO_3$ and KOH electrolytes at different pH (pH = 8, 10, 11, 12, 13, and 14) at a current density of 100 mA cm^{-2}. Error bars correspond to the standard deviation of five independent measurements.

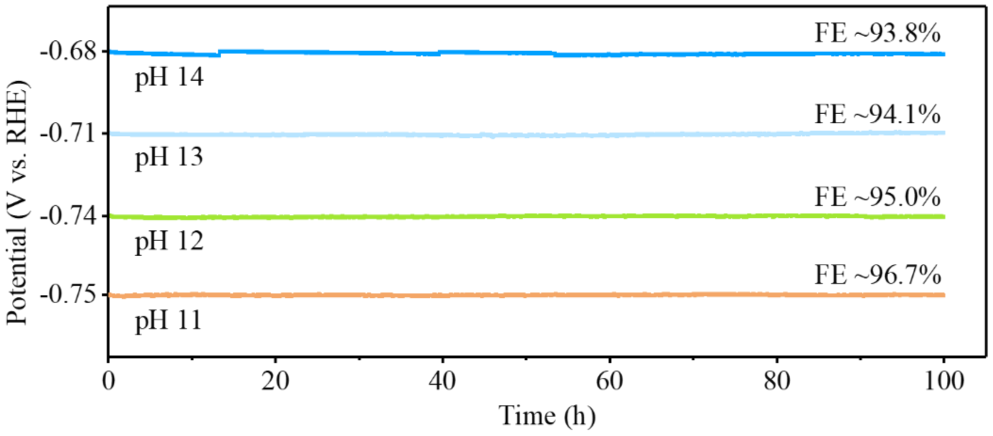

Supplementary Figure 28. The CO_2R performance of $Bi_{0.1}Sn$ catalysts with the carbon NPs and graphite coatings in 1 M $KHCO_3$ and KOH electrolytes at different pH at an applied current density of 100 mA cm^{-2}.

.

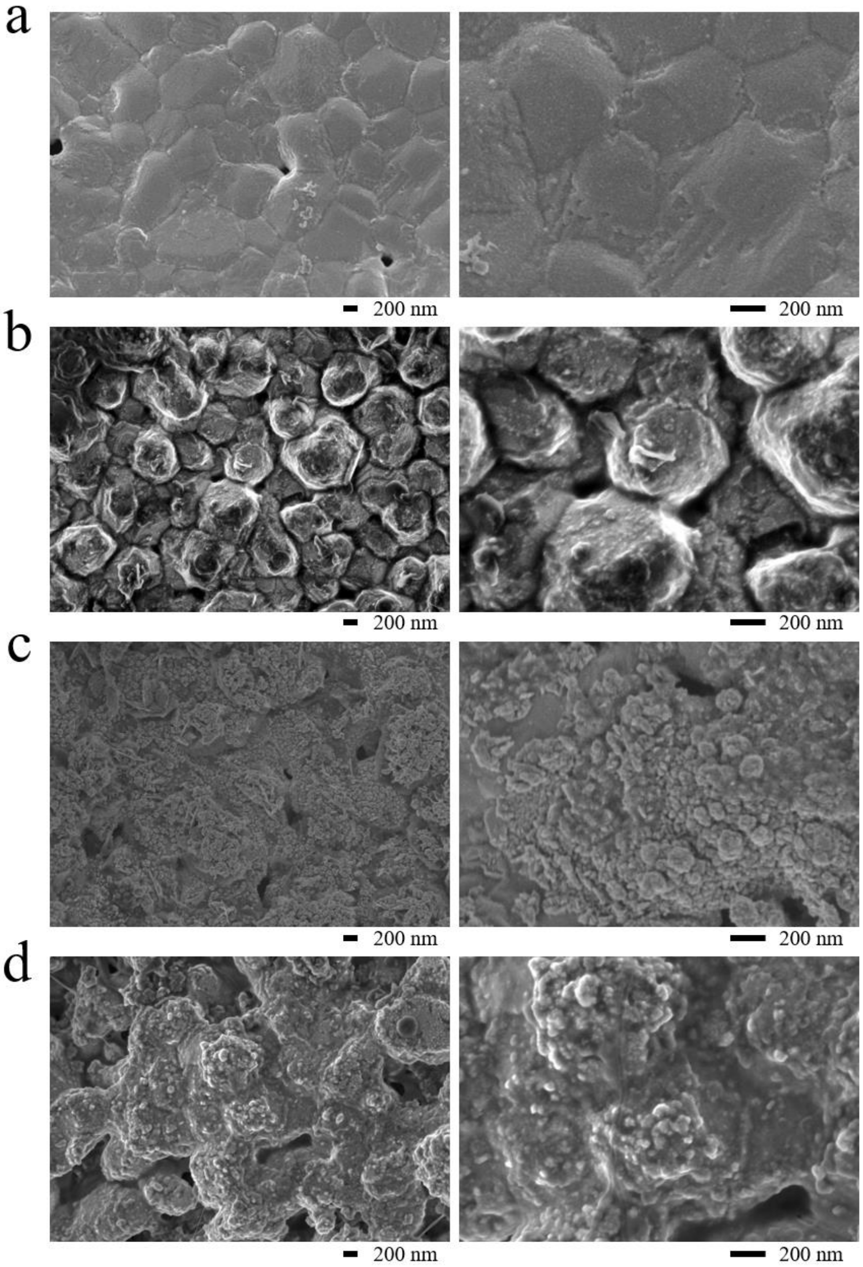

Supplementary Figure 29. SEM images of $Bi_{0.1}Sn$ after 100-hour tests in 1 M $KHCO_3$ and KOH electrolytes at different pH at an applied current density of 100 mA cm^{-2}. **a,** At pH 11. **b,** At pH 12. **c,** At pH 13. **d,** At pH 14.

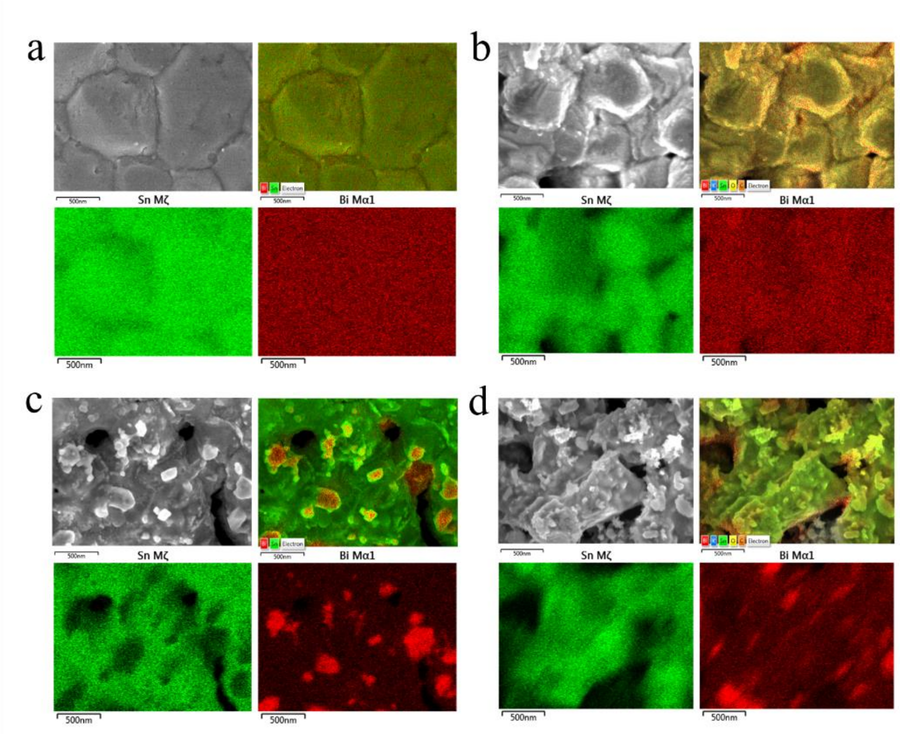

Supplementary Figure 30. SEM-EDX of $Bi_{0.1}Sn$ after 100-hour tests in 1 M $KHCO_3$ and KOH electrolytes at different pH at an applied current density of 100 mA cm^{-2}. **a,** At pH 11. **b,** At pH 12. **c,** At pH 13. **d,** At pH 14.

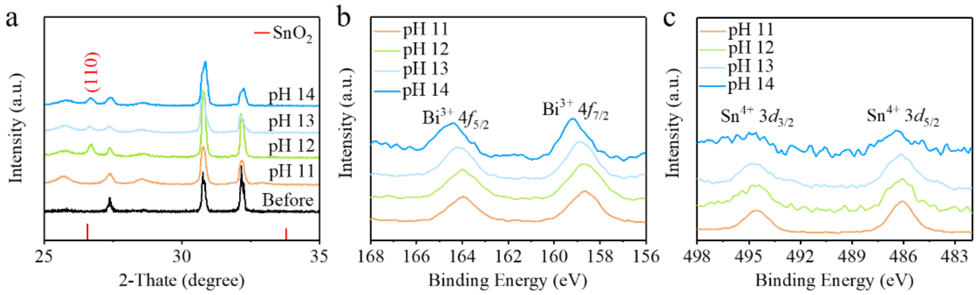

Supplementary Figure 31. **a,** XRD and **b, c,** XPS results of $Bi_{0.1}Sn$ after 100-hour tests in 1 M $KHCO_3$ and KOH electrolytes at different pH at an applied current density of 100 mA cm^{-2}.

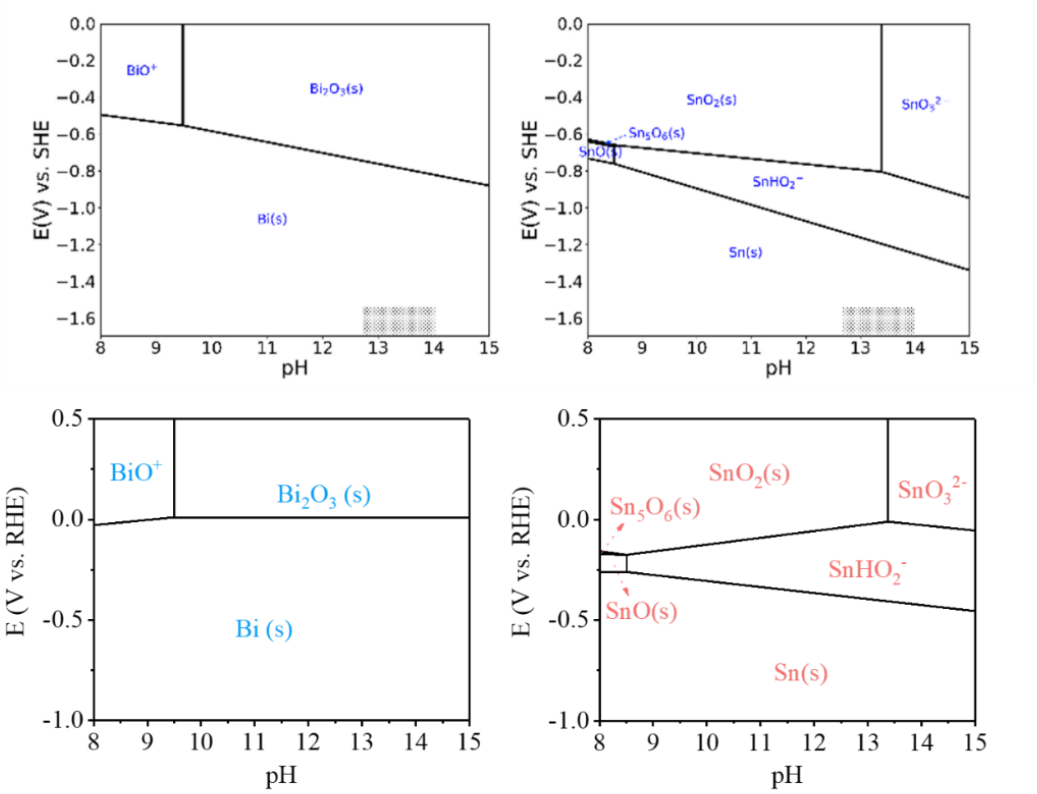

Supplementary Figure 32. Pourbaix diagram of Bi and Sn.

Note that the amount of catalyst used for the electrochemical test is ~650 μg (area: 1 cm^2, thickness: 700 nm), the overall amount of electrolyte used for the stability test is over 1 kilogram (> 1 litre). We confirm catalyst loading by inductively coupled plasma atomic emission spectroscopy (ICP-AES) analysis. We first completely dissolved different sizes of $Bi_{0.1}Sn$ (1 cm^2, 4 cm^2 and 15 cm^2) in the HCl solutions. We then determined the amounts of dissolved Bi and Sn by ICP-AES (Supplementary Figure 33).

We performed CO_2R stability test with $Bi_{0.1}Sn$ over 100 hours. It indicates that the catalyst is mostly stable at negative protecting potentials in the Pourbaix diagram. However, we need to be careful with the unexpected corrosion that might randomly occur on the catalyst surfaces during the long-term operation. Therefore, it is important to lower the electrolyte pH for achieving long stability in combination with high selectivity and energy efficiency.

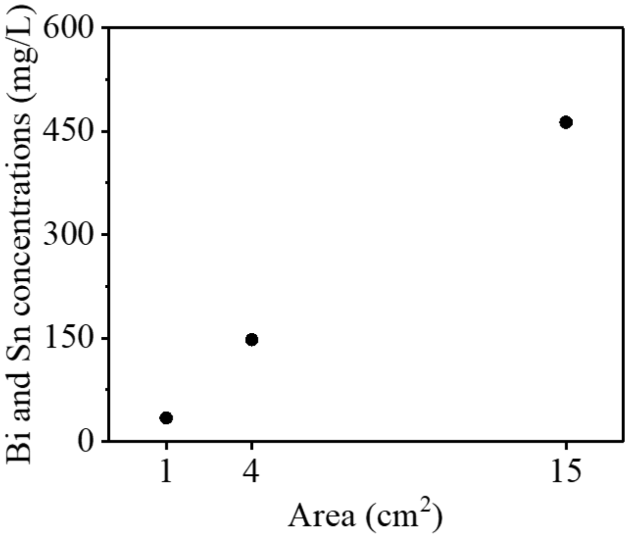

Supplementary Figure 33. ICP-AES analysis of the total concentrations of Bi and Sn in the HCl solution with different catalyst sizes (1 cm^2, 4 cm^2 and 15 cm^2).

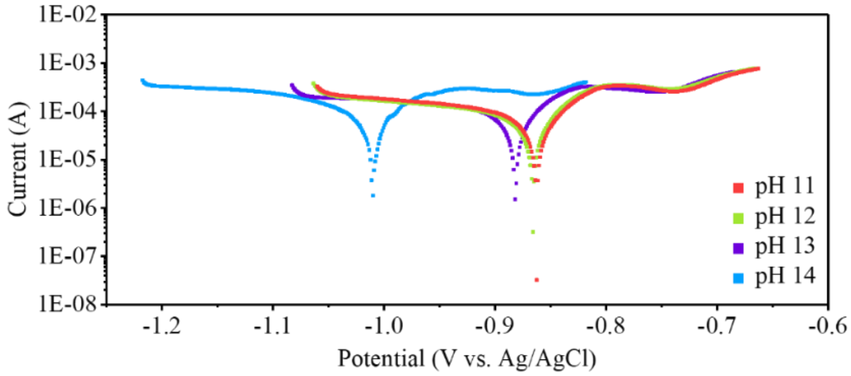

Supplementary Figure 34. Linear polarization curves of the $Bi_{0.1}Sn$ catalyst of 1 M $KHCO_3$ and KOH electrolyte solution at different pH.

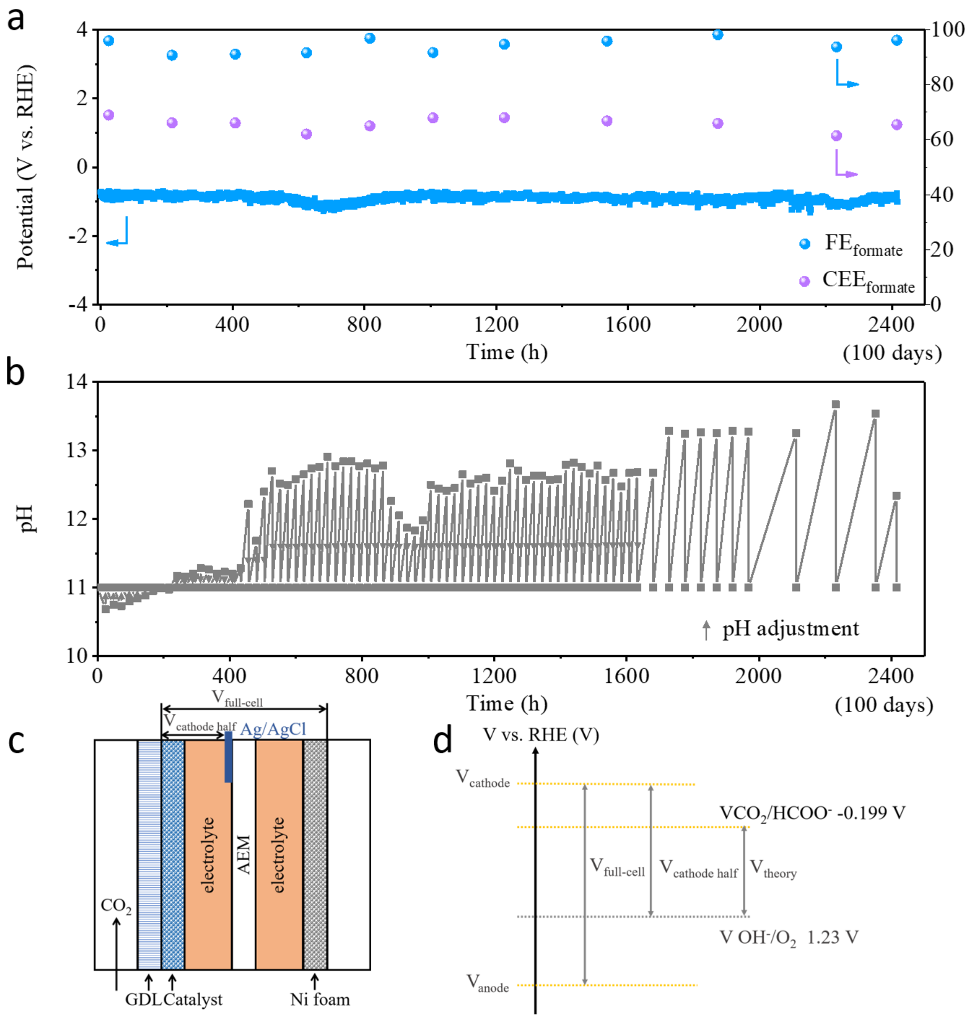

Supplementary Figure 35. **a**, The CO_2R chronopotentiometry curve (blue line), CO_2-to-formate FE (blue dots), and CO_2-to-formate CEE (purple dots) in a 1 M $KHCO_3$ and KOH electrolytes at pH = 11 at an applied current density of 100 mA cm^{-2}. **b**, The corresponding pH adjustment process within 2400 hours. **c**, Schematic of a full-cell CO_2R setup. **d**, Potential diagram to calculate the full-cell energy efficiency (EE) when using nickel foam on the anode side.

We performed CO_2R stability tests for the $Bi_{0.1}Sn$ catalyst at pH 11. We changed electrolytes every 48 hours to address the potential crossover of electrolytes through membranes, and to reduce the carbonate precipitates on the backside of the gas diffusion layer. We found that the electrolyte pH varied during CO_2R. This is likely related to the combined effect of (i) OH^- ions reacting with CO_2 to form HCO_3^-, decreasing the electrolyte pH and (ii) H^+ ions reacting with CO_2 to form $HCOO^-$ to increase the electrolyte pH. The above competition results in a variation of electrolyte surface pH. For long operation at pH 11, we calibrated the electrolyte pH to 11 every 48 hours. Our recorded pH graphs can be found in Supplementary Fig. 35b. We measured the full-cell voltage of 4.0–4.3 V for the flow-cell device with $Bi_{0.1}Sn$ catalysts as the cathode and an unmodified commercial Ni foam as the anode. We achieved a 35 ± 2% full-cell EE when coupled with a commercial nickel foam (not the best-performance anode) on the anode side.

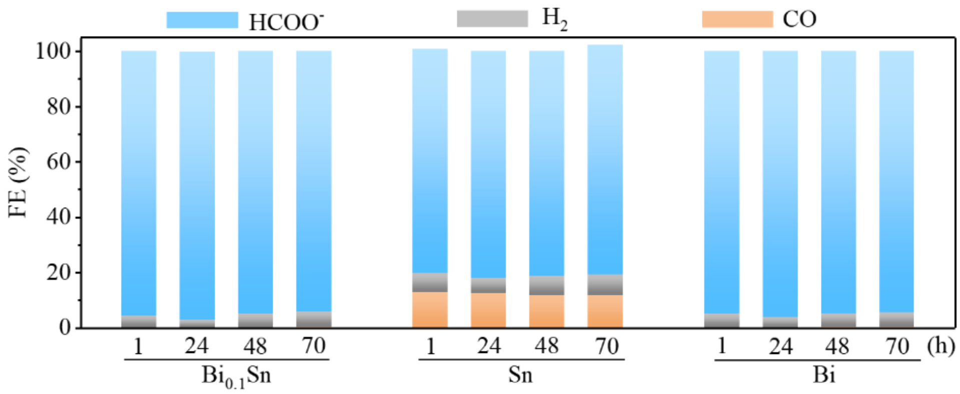

Supplementary Figure 36. FE of $HCOO^-$, H_2 and CO with $Bi_{0.1}Sn$, Sn and Bi catalysts in 1 M $KHCO_3$ and KOH electrolyte at pH 11 at an applied current density of 100 mA cm^{-2}.

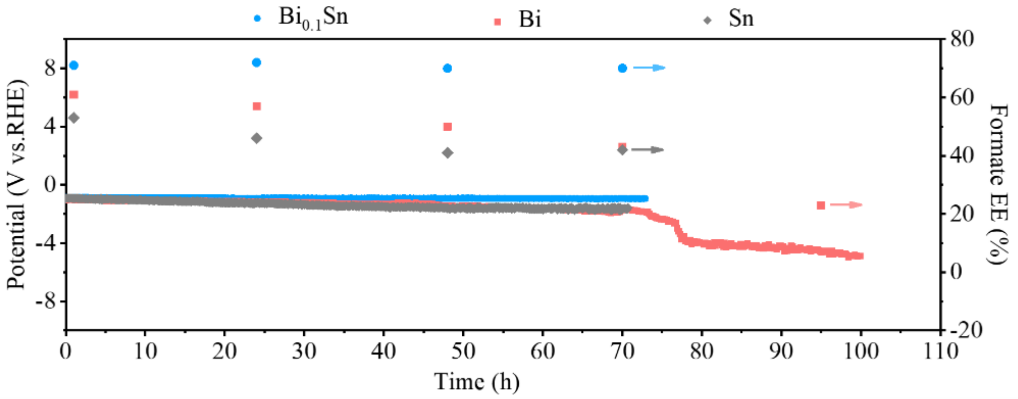

Supplementary Figure 37. The CO_2-reduction chronopotentiometry curve and CO_2-to-formate CEE of $Bi_{0.1}Sn$, Bi and Sn catalysts in a 1 M $KHCO_3$ and KOH electrolytes at pH = 11 at an applied current density of 100 mA cm^{-2}.

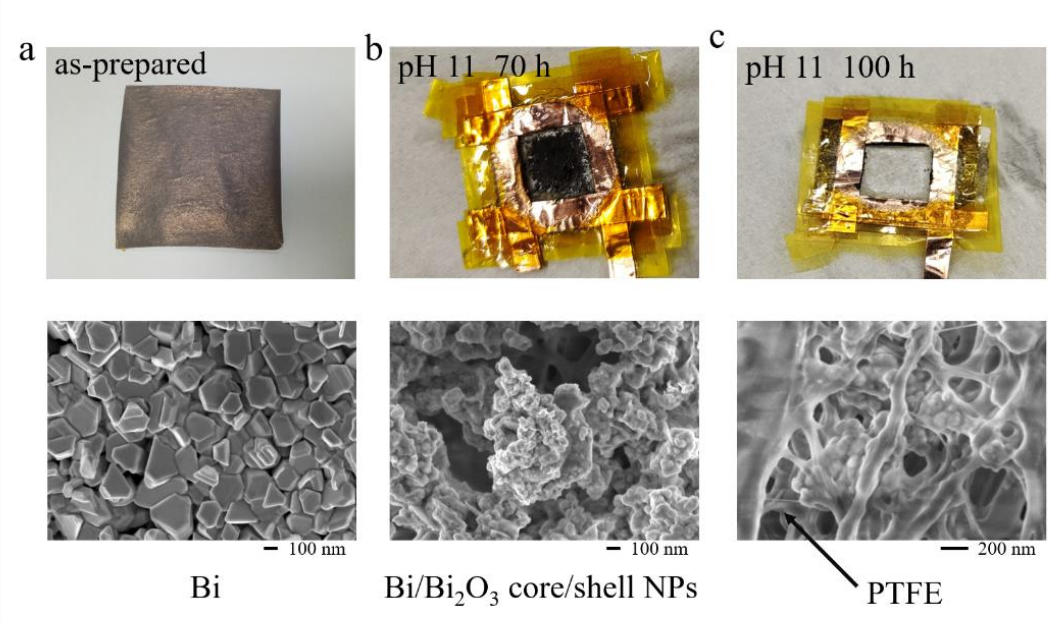

Supplementary Figure 38. Optical images and SEM images of the Bi catalyst after 70 or 100 hours of reaction in 1 M $KHCO_3$ and KOH electrolytes at pH 11. **a,** As-prepared Bi. **b,** After 70 hours of reaction. **c,** After 100 hours of reaction.

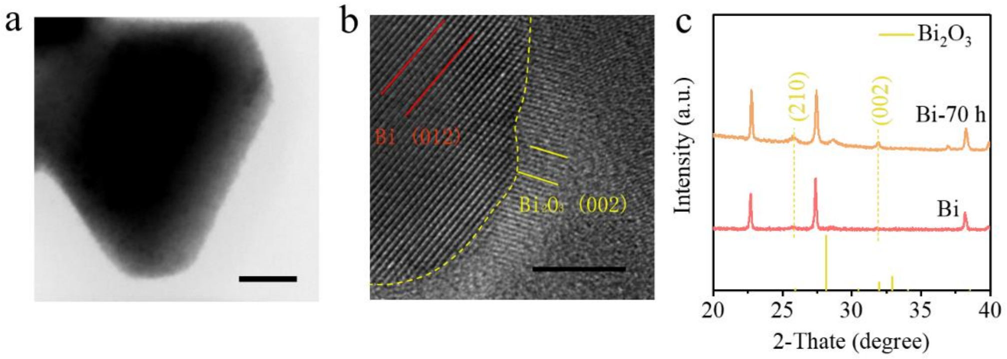

Supplementary Figure 39. TEM, HRTEM and XRD data of the Bi catalyst after 70 hours of reaction in 1 M $KHCO_3$ and KOH electrolytes at pH 11. **a**, TEM image; the scale bar is 20 nm. **b**, HRTEM image; the scale bar is 5 nm. **c,** XRD patterns of the electrode before (red) and after (orange) the reaction.

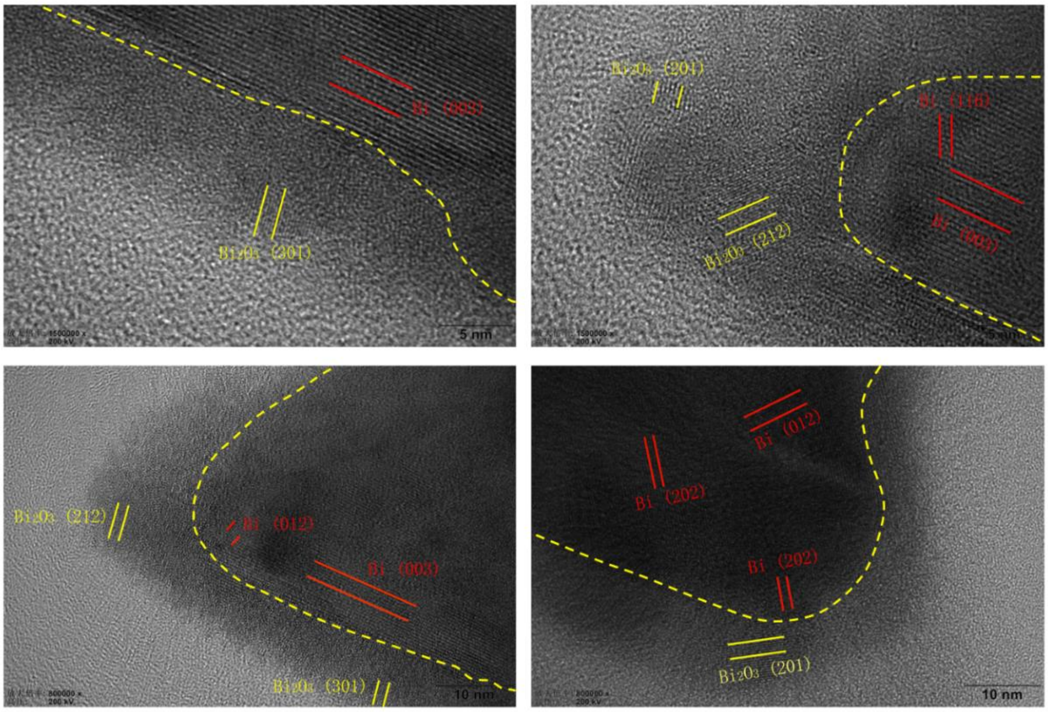

Supplementary Figure 40. HRTEM images of the Bi catalyst after 70 hours of reaction in 1 M $KHCO_3$ and KOH electrolytes at pH 11. A clear Bi/Bi_2O_3 core/shell structure is observed. The yellow line indicates the boundary between Bi and Bi_2O_3.

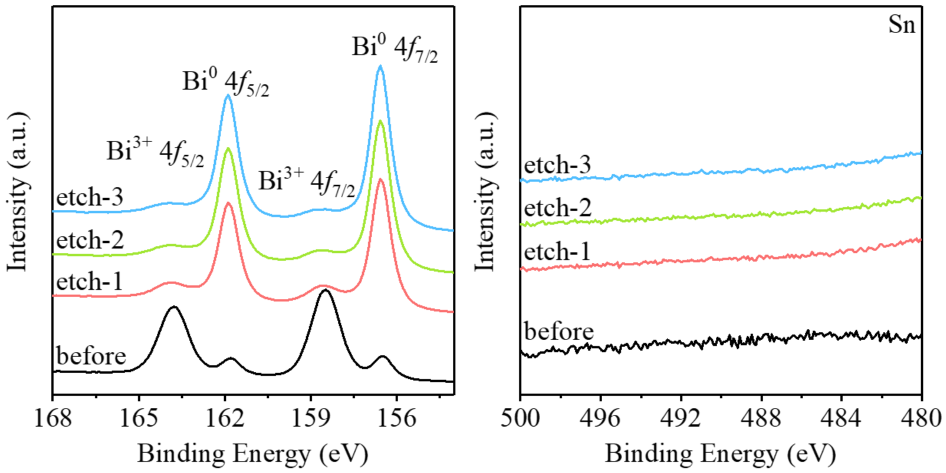

Supplementary Figure 41. XPS depth profiles of the Bi electrode before the CO_2R reaction. The black, red, green and blue lines represent the data before etching (black) and after different time courses of soft Ar etching (red, green, blue), respectively.

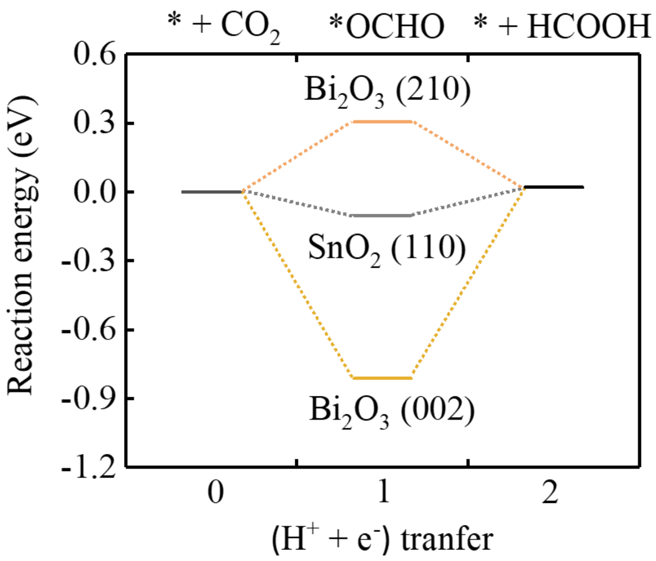

Supplementary Figure 42. Reaction energy of formate formation on Bi_2O_3 (210), Bi_2O_3 (002), and $Bi_{0.1}Sn/SnO_2$ composite with SnO_2 (110) on top without applying any external potential (U = 0 eV).

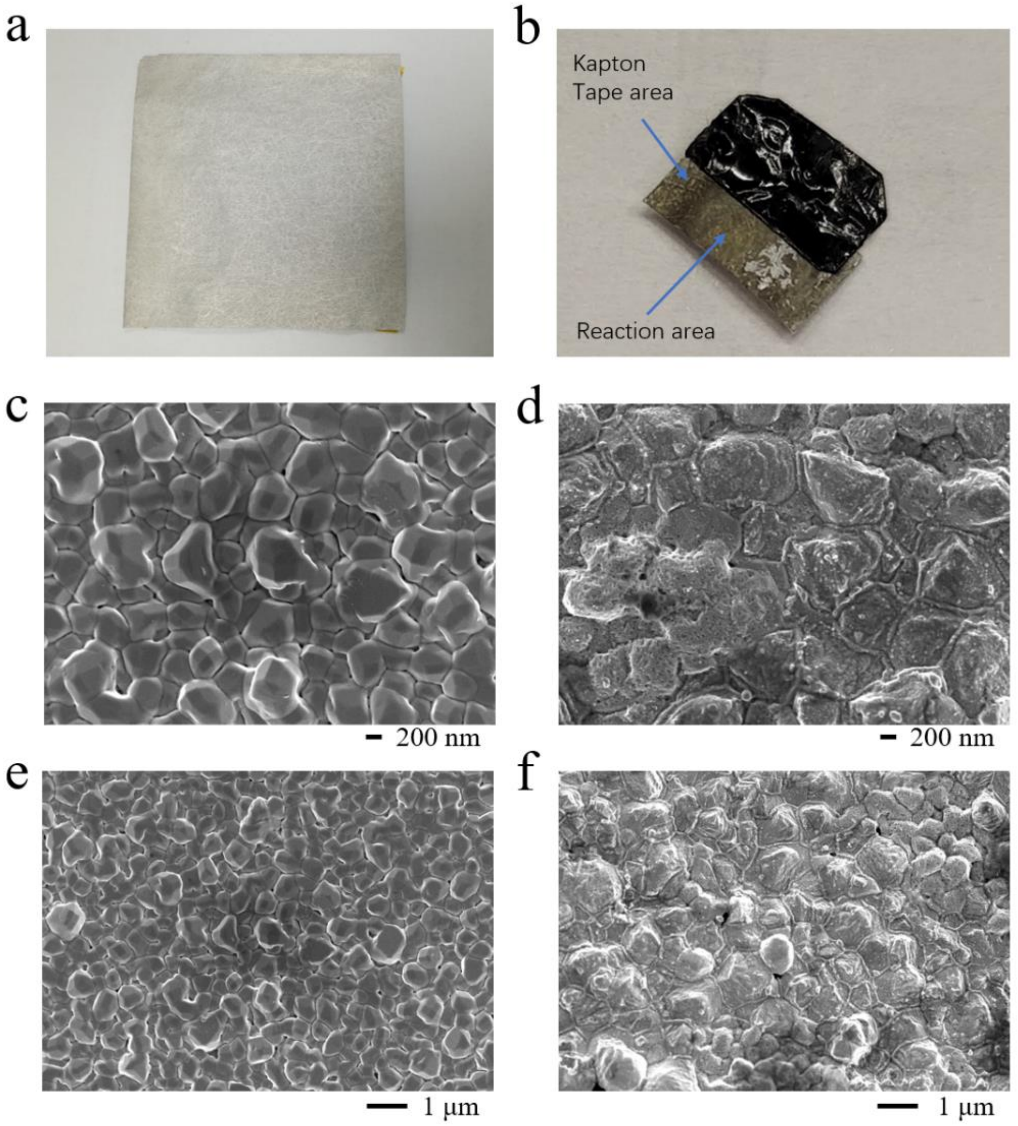

Supplementary Figure 43. The $Bi_{0.1}Sn$ catalyst after 70 hours of reaction in 1 M $KHCO_3$ and KOH electrolytes at pH 11. **a,** Optical image, **c, e,** SEM image of as-prepared $Bi_{0.1}Sn$. **b,** Optical image, **d, f,** SEM image of $Bi_{0.1}Sn$ after 70 hours of reaction.

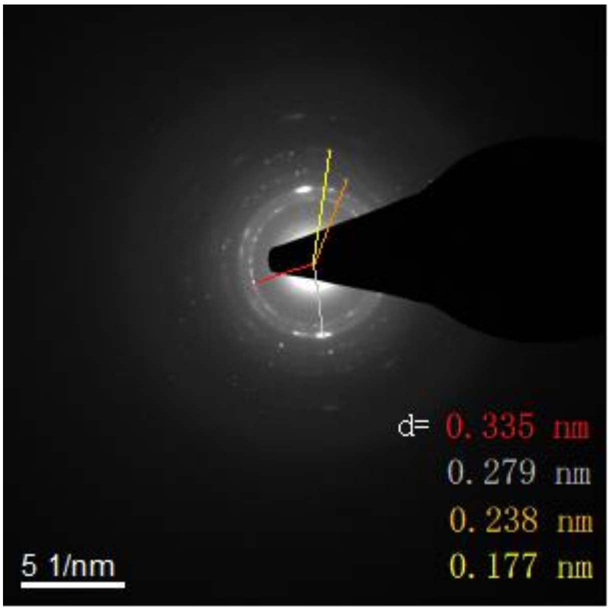

Supplementary Figure 44. SAED pattern of the $Bi_{0.1}Sn$ catalyst after 70 hours of reaction in 1 M $KHCO_3$ and KOH electrolytes at pH 11.

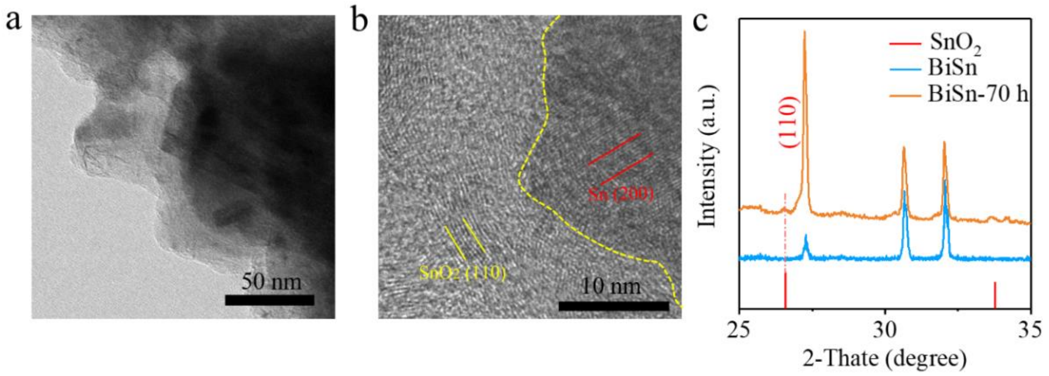

Supplementary Figure 45. TEM, HRTEM and XRD data of the $Bi_{0.1}Sn$ catalyst after 70 hours of reaction in 1 M $KHCO_3$ and KOH electrolytes at pH 11. **a**, TEM image. **b**, HRTEM image. **c,** XRD patterns of the electrode before (blue) and after (orange) the reaction.

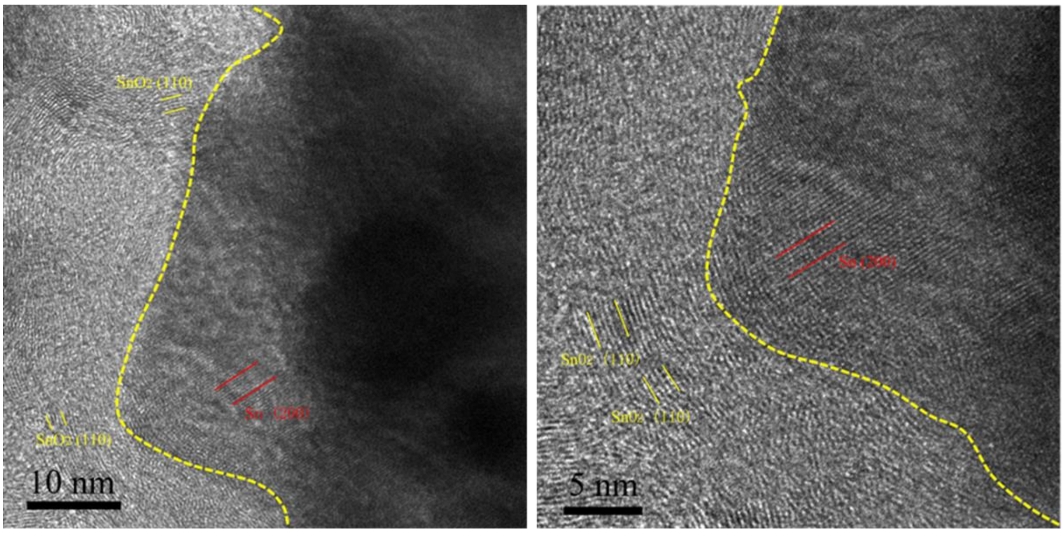

Supplementary Figure 46. HRTEM images of the $Bi_{0.1}Sn$ catalyst after 70 hours of reaction in 1 M $KHCO_3$ and KOH electrolytes at pH 11. A clear SnO_2 layer is observed. The yellow line indicates the boundary between BiSn and SnO_2.

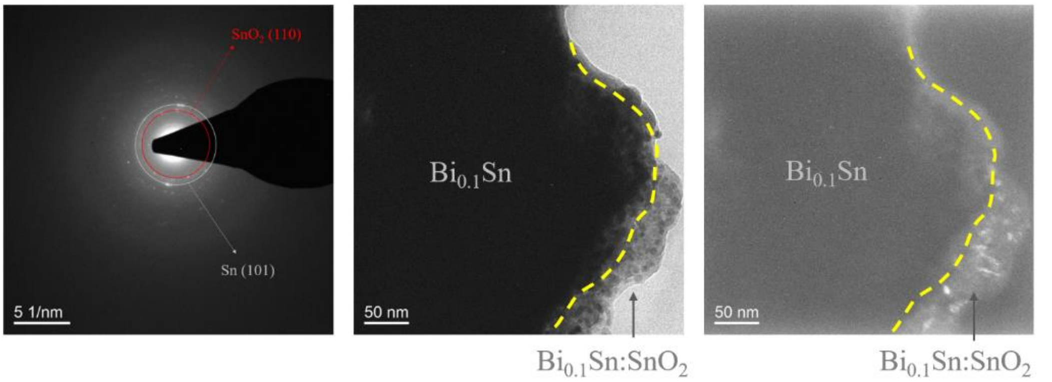

Supplementary Figure 47. SAED pattern as well as bright-field and dark-field TEM images of the $Bi_{0.1}Sn$ catalyst after 70 hours of reaction in 1 M $KHCO_3$ and KOH electrolytes at pH 11.

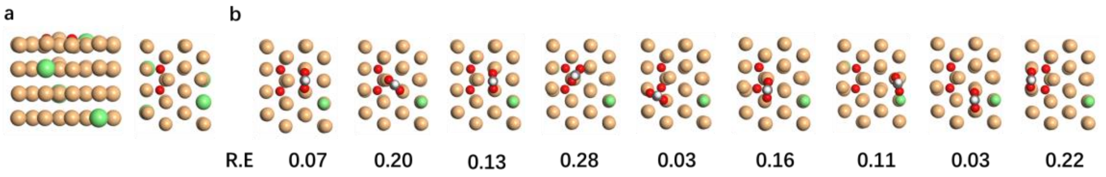

Supplementary Figure 48. DFT calculations of *OCHO on 4Bi/64Sn (200)-2O surfaces. **a,** Side (left) and top (right) views of the slab model with 1 Bi atom at each layer of the Sn (200)-2O surface in the absence of OCHO. The "Surface-1" model in Figure S14 was used as the initial configuration to build the model. An additional two oxygen atoms were added to form the Sn-O covalent bond at the surface. Bi, Sn, O, C and H atoms are represented by green, brown, red, grey and white spheres, respectively, and the size of the Bi atoms is slightly enlarged for display. **b,** Different configurations and the corresponding reaction energy (in eV) of *OCHO bound to the 4Bi/64Sn (200)-2O structures.

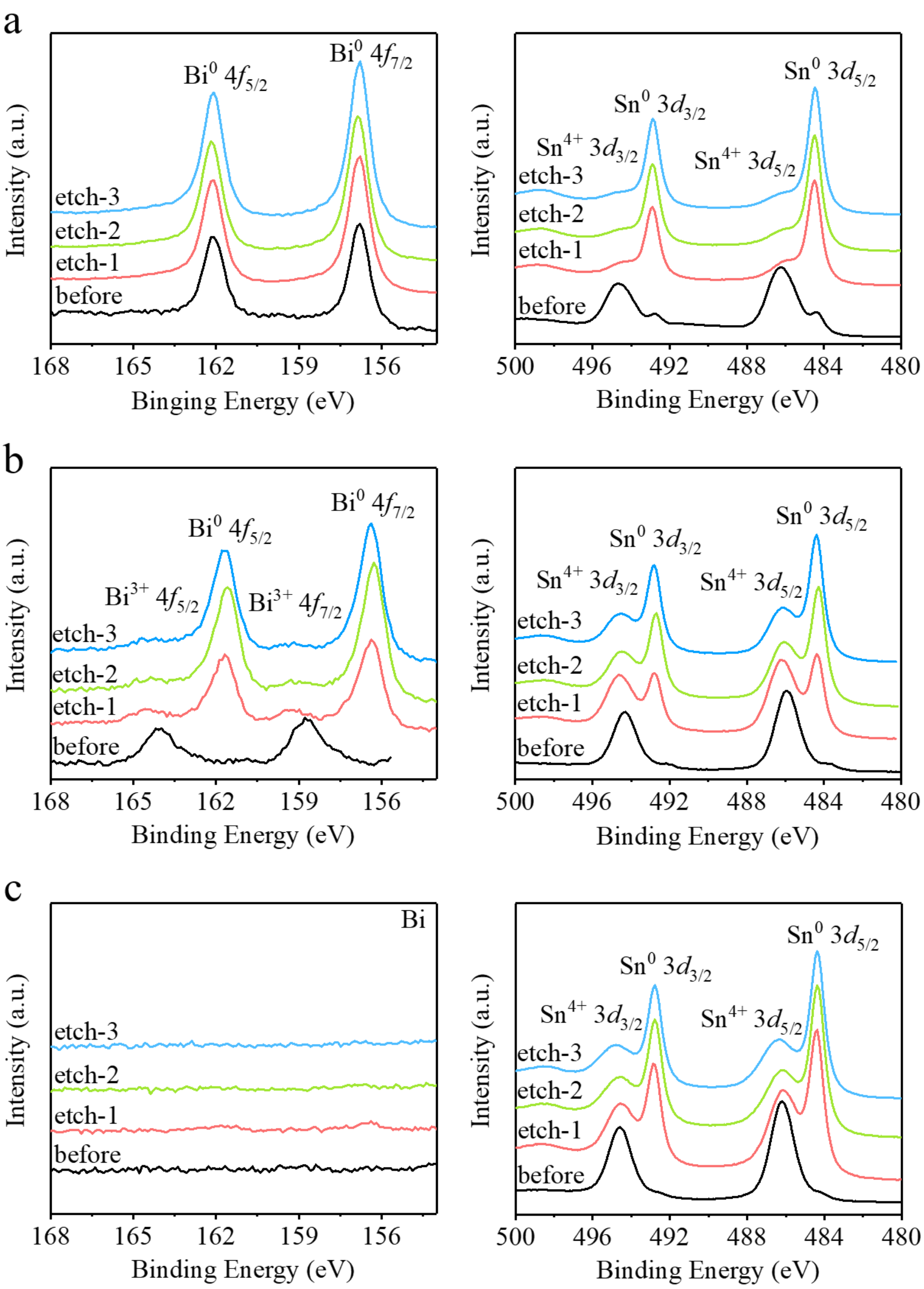

Supplementary Figure 49. XPS depth profiles of **a,** $Bi_{0.1}Sn$ electrode before the CO_2R reaction, **b,** post-reaction $Bi_{0.1}Sn$ electrode after the 100-hour CO_2R reaction at 100 mA cm^{-2} in 1 M $KHCO_3$ and KOH electrolytes at pH = 11, **c,** post-reaction Sn after 70-hour CO_2 reduction at 100 mA cm^{-2} in 1 M $KHCO_3$ and KOH electrolytes at pH = 11. The black, red, green and blue lines represent the data before etching (black) and after different time courses of soft Ar etching (red, green, blue), respectively. The etched depth in each soft Ar etching is ~3–5 nm.

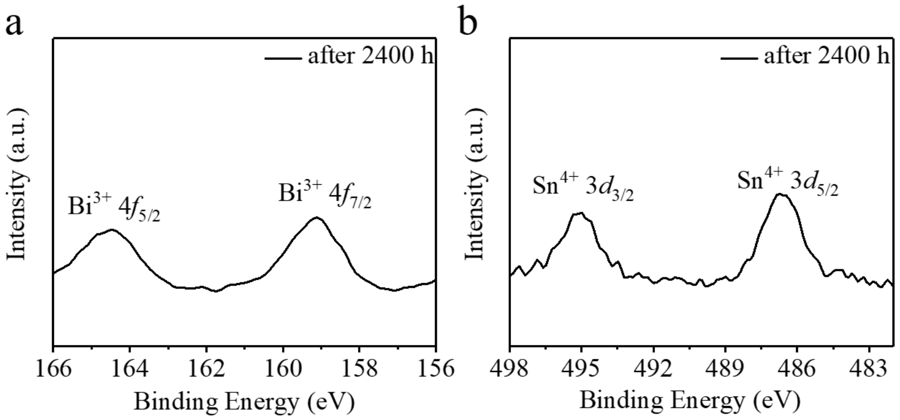

Supplementary Figure 50. XPS spectra of $Bi_{0.1}Sn$ catalyst after CO_2 reduction at 100 mA cm^{-2} in 1 M $KHCO_3$ and KOH electrolytes at pH = 11 after reaction, confirming that Bi and Sn signals are readily observed on the electrode surfaces.

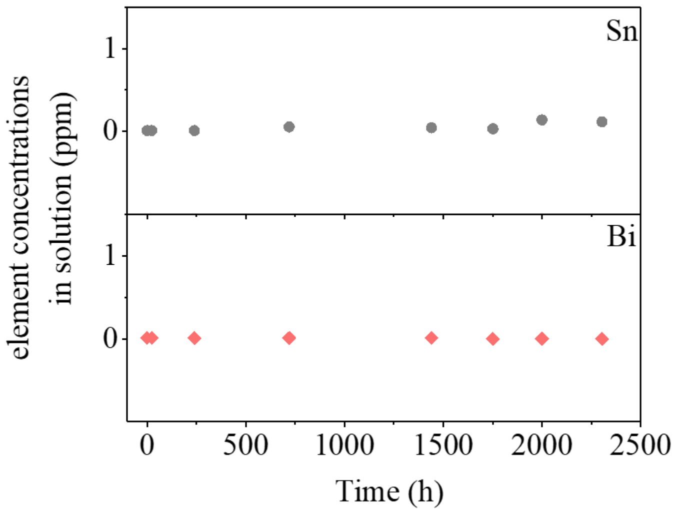

Supplementary Figure 51. ICP-AES analysis results of Bi and Sn ions in the testing solution with different operational time. The $Bi_{0.1}Sn$ catalysts on PTFE were measured at 100 mA cm^{-2} in 1 M $KHCO_3$ and KOH electrolytes at pH = 11 for 2400 hours. There is no obvious leaching of Bi and Sn into the solution after the 2400-hour test.

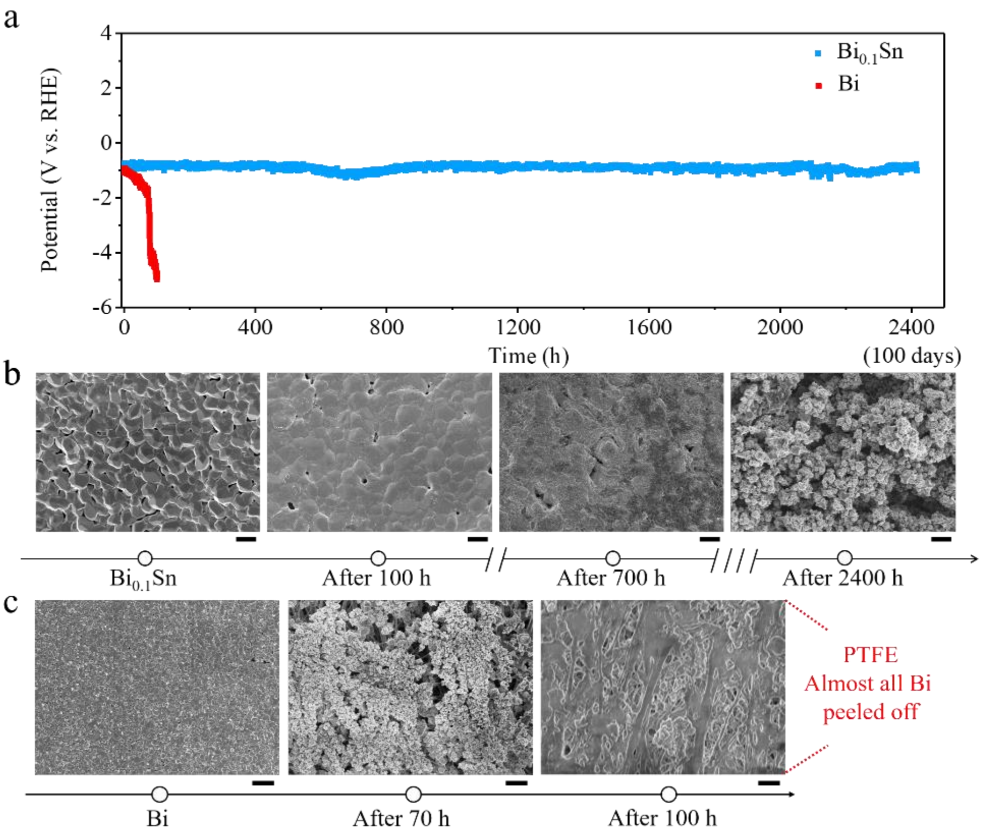

Supplementary Figure 52. (a) The CO_2-reduction chronopotentiometry curve of $Bi_{0.1}Sn$ and Bi catalysts in the 1 M $KHCO_3$ and KOH electrolytes at pH 11 at an applied current density of 100 mA cm^{-2}. The SEM images of (b) $Bi_{0.1}Sn$ and (c) Bi after different time courses of the stability tests. The scale bars in the SEM images are 1 μm.

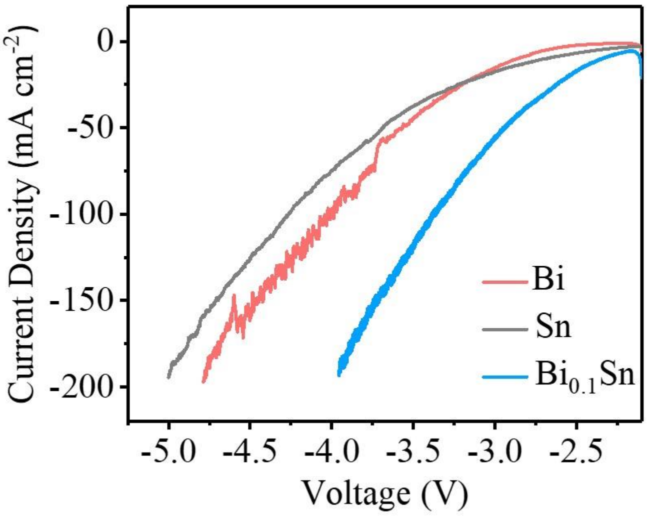

Supplementary Figure 53. LSV curves of Bi, Sn and $Bi_{0.1}Sn$ in an AEM-based MEA system.

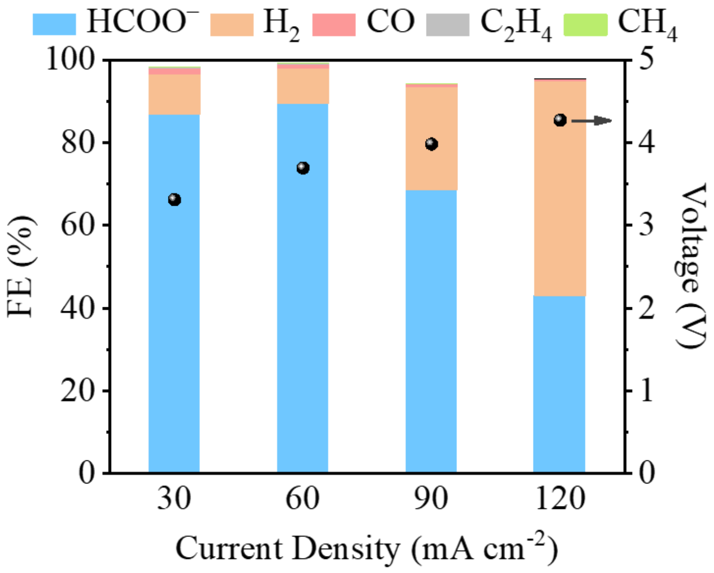

Supplementary Figure 54. The selectivity of $Bi_{0.1}Sn$ at different current densities in 0.1 M $KHCO_3$ and corresponding voltages in a solid-state CEM-based MEA system.

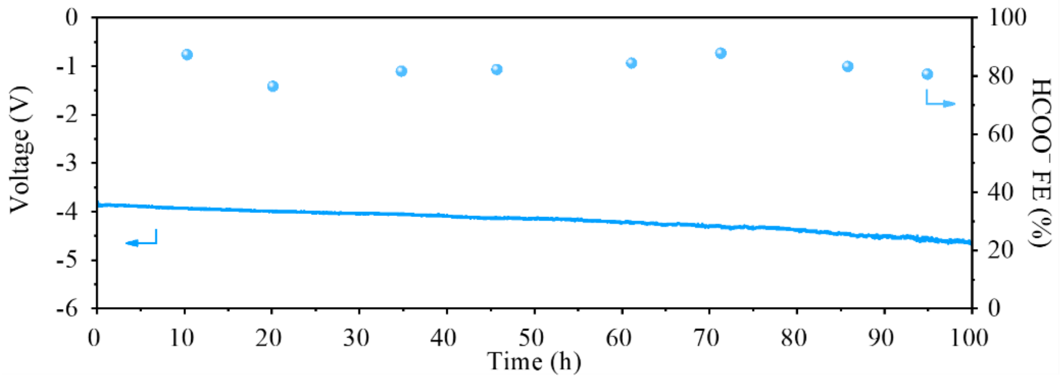

Supplementary Figure 55. The CO_2R chronopotentiometry curve (blue line), and CO_2-to-$HCOO^-$ FE (blue dots) in 0.1 M $KHCO_3$ electrolyte at an applied current density of 60 mA cm^{-2}. The full-cell potentials in the MEA tests are presented without IR correction. The catholyte pH is 7.2 in our CEM based MEA system.

Supplementary Table 1. The gas phase molecules and their thermodynamic quantities (eV).

Molecule	EDFT	ZPE	∫CvdT	-TS	G
CO_2	-22.95	0.31	0.10	-0.66	-22.79
H_2	-6.77	0.27	0.09	-0.43	-6.94
CO	-14.78	0.13	0.09	-0.67	-15.22
HCOOH	-29.87	0.89	0.11	-1.05	-29.71

Supplementary Table 2. The binding and reaction energies of the different catalyst systems. For each system, different configurations of *OCHO were used to get the average value.

System	Binding energy（eV）	Reaction energy（eV）
Sn (101)	-0.526	0.295
Sn (200)	-0.412	0.218
1Bi/64Sn-Sn (200)	-0.326 ± 0.010	0.132 ± 0.010
2Bi/64Sn-Sn (200)	-0.340 ± 0.007	0.149 ± 0.011
4Bi/64Sn-Sn (200)	-0.316 ± 0.013	0.115 ± 0.014
8Bi/64Sn-Sn (200)	-0.330 ± 0.010	0.117± 0.051
4Bi/64Sn-2O-Sn (200)	-0.307 ± 0.046	0.136 ± 0.029
Bi (003)	-0.036	0.206
Bi (012)	0.123	0.319

Supplementary Table 3. The potential of Bi, $Bi_{0.1}Sn$ and Sn at different current density in 1 M KOH electrolyte.

Catalyst	Current Density	Potential (V vs. RHE)
	100 mA cm^{-2}	-0.68
Bi0.1Sn	200 mA cm^{-2}	-0.75
	300 mA cm^{-2}	-0.90
	100 mA cm^{-2}	-0.83
Bi	200 mA cm^{-2}	-1.10
	300 mA cm^{-2}	-1.70
	100 mA cm^{-2}	-0.90
Sn	200 mA cm^{-2}	-1.30
	300 mA cm^{-2}	-1.70

Supplementary Table 4. A comparison of this work with previously reported data.

Materials	Stability (h)	Potential (V_{RHE})	J (mA/cm^2)	FE_{HCOOH}	CEE_{HCOOH}	Cell	Reference
$Bi_{0.1}Sn$	2400	-0.68-0.75	100	95%-98%	70%-75%	Flow-cell	This work
Bi dendrite	12	-0.73	3	89%	65%	H-cell	[14]
nano-SnO_2	18	-1.11	8	86%	53%	H-cell	[15]
Sn/SnS_2	12	-0.74	14	84%	61%	H-cell	[16]
Bi NSs	12	-0.8	5	99%	70%	H-cell	[17]
Bi NSs	10	-0.88	15-16	95%	64%	H-cell	[18]
Ag-Sn core-shell NPs	25	-0.8	16	80%	56%	H-cell	[19]
Sn quantum sheets	50	-1.1	22	85%	52%	H-cell	[20]
POD-Bi	0.45	-0.48*	100	93%	79%	Flow-cell	[21]
SnO_2 on Carbon cloth	24	-0.98	50	87%	56%	H-cell	[22]
nanotube-derived Bi	13	-0.85	140	95%-98%	65%	Flow-cell	[7]
2D-Bi	100	/	30	80%	Not reported		[23]
$Sn\text{-}Cu/SnO_x$ core-shell NPs	40	-0.55	243.1	90%	72%	Flow-cell	[24]
Bi NPs on Sn NSs with Bi-Sn interfaces	100	-1.14	58	91%	55%	H-cell	[25]
Phase-segregated Bi_xSn_{1-x} ($x = 0.2 - 0.8$)	1	-1.1	10	78%	48%	H-cell	[26]
Bi@Sn core-shell NPs	20	-1.1	31	91%	56%	H-cell	[27]
$Sn_{0.80}Bi_{0.20}$@$Bi\text{-}SnO_x$	50	-0.88	21.8	95.8%	65%	Flow-cell	[28]

Supplementary Table 5. Performance of Bi, Sn, and $Bi_{0.1}Sn$ catalysts in AEM- and solid-state CEM-based MEA systems.

Materials, AEM or CEM, J (mA cm^{-2}), full-cell V (V)	$HCOO^-$	H_2	CO	C_2H_4	CH_4	Overall
Bi, AEM, 30, 3.32	89.97%	10.21%	1.73%	0.27%	0.09%	102.27%
Bi, AEM, 60, 3.57	90.42%	7.67%	1.82%	0.35%	0.07%	100.33%
Bi, AEM, 90, 3.89	92.26%	5.26%	1.33%	0.28%	0.05%	99.18%
Bi, AEM, 120, 4.09	95.84%	6.01%	0.61%	0.08%	0.06%	102.60%
Bi, AEM, 180, 4.45	77.08%	17.27%	0.91%	0.08%	0.56%	95.90%
Sn, AEM, 30, 3.33	91.13%	6.91%	1.92%	0.41%	0.19%	100.56%
Sn, AEM, 60, 3.61	92.87%	5.69%	1.58%	0.32%	0.13%	100.59%
Sn, AEM, 90, 3.96	92.51%	4.68%	1.19%	0.19%	0.08%	98.65%
Sn, AEM, 120, 4.29	93.54%	5.42%	0.46%	0.11%	0.03%	99.56%
Sn, AEM, 180, 4.67	73.7%	21.61%	0.09%	0.06%	0.18%	95.64%
$Bi_{0.1}Sn$, AEM, 30, 3.05	89.97%	9.07%	2.13%	0.32%	0.15%	101.64%
$Bi_{0.1}Sn$, AEM, 60, 3.22	92.27%	7.24%	1.78%	0.26%	0.11%	101.66%
$Bi_{0.1}Sn$, AEM, 90, 3.36	87.85%	6.13%	1.42%	0.18%	0.08%	95.66%
$Bi_{0.1}Sn$, AEM, 120, 3.66	97.81%	3.12%	0.86%	0.09%	0.02%	101.90%
$Bi_{0.1}Sn$, AEM, 180, 4.06	92.82%	5.07%	0.52%	0.05%	0.01%	98.47%
$Bi_{0.1}Sn$, CEM, 30, 3.31	86.79%	9.87%	1.29%	0.23%	0.07%	98.25%
$Bi_{0.1}Sn$, CEM, 60, 3.69	89.38%	8.69%	0.88%	0.17%	0.04%	99.16%
$Bi_{0.1}Sn$, CEM, 90, 3.98	68.56%	24.91%	0.61%	0.09%	0.02%	94.19%
$Bi_{0.1}Sn$, CEM, 120, 4.27	43.07%	51.91%	0.34%	0.04%	0.01%	95.37%

Supplementary References

1. Chen, BB. et al. Indented Cu_2MoS_4 nanosheets with enhanced electrocatalytic and photocatalytic activities realized through edge engineering. *Phys. Chem. Chem. Phys.* **18,** 6713-6721 (2016).
2. Lee, JA. & Raynor, GV. The Lattice Spacings of Binary Tin-Rich Alloys. *Proceedings of the Physical Society Section B* **67,** 737-747 (1954).
3. Kresse, G. & Hafner, J. Ab initio molecular-dynamics simulation of the liquid-metal--amorphous-semiconductor transition in germanium. *Phys. Rev. B* **49,** 14251-14269 (1994).
4. Blöchl, PE. Projector augmented-wave method. *Phys. Rev. B* **50,** 17953-17979 (1994).
5. Kresse, G. & Joubert, D. From ultrasoft pseudopotentials to the projector augmented-wave method. *Phys. Rev. B* **59,** 1758-1775 (1999).
6. Perdew, JP., Burke, K. & Ernzerhof, M. Generalized Gradient Approximation Made Simple. *Phys. Rev. Lett.* **77,** 3865-3868 (1996).
7. Gong, Q. et al. Structural defects on converted bismuth oxide nanotubes enable highly active electrocatalysis of carbon dioxide reduction. *Nat. Commun.* **10,** 2807 (2019).
8. Klinkova, A. et al. Rational Design of Efficient Palladium Catalysts for Electroreduction of Carbon Dioxide to Formate. *ACS Catal.* **6,** 8115-8120 (2016).
9. Yoo, JS. et al. Theoretical Insight into the Trends that Guide the Electrochemical Reduction of Carbon Dioxide to Formic Acid. *ChemSusChem* **9,** 358-363 (2016).
10. Christensen, R., Hansen, HA. & Vegge, T. Identifying systematic DFT errors in catalytic reactions. *Catal. Sci. Technol.* **5,** 4946-4949 (2015).
11. Kresse, G. & Furthmüller, J. Efficient iterative schemes for ab initio total-energy calculations using a plane-wave basis set. *Phys. Rev. B* **54,** 11169-11186 (1996).
12. Nørskov, JK. et al. Origin of the Overpotential for Oxygen Reduction at a Fuel-Cell Cathode. *J. Phys. Chem. B* **108,** 17886-17892 (2004).
13. Ercelik, M., Ozden, A., Devrim, Y. & Colpan, CO. Investigation of Nafion based

composite membranes on the performance of DMFCs. *Int. J. Hydrogen Energy* **42,** 2658-2668 (2017).

14. Koh, JH. et al. Facile CO_2 Electro-Reduction to Formate via Oxygen Bidentate Intermediate Stabilized by High-Index Planes of Bi Dendrite Catalyst. *ACS Catal.* **7,** 5071-5077 (2017).

15. Zhang, S., Kang, P. & Meyer, TJ. Nanostructured tin catalysts for selective electrochemical reduction of carbon dioxide to formate. *J. Am. Chem. Soc.* **136,** 1734-1737 (2014).

16. Li, F. et al. Towards a better Sn: Efficient electrocatalytic reduction of CO_2 to formate by Sn/SnS_2 derived from SnS_2 nanosheets. *Nano Energy* **31,** 270-277 (2017).

17. Yang, H. et al. Selective CO_2 Reduction on 2D Mesoporous Bi Nanosheets. *Adv. Energy Mater.* **8,** 1801536 (2018).

18. Han, N. et al. Ultrathin bismuth nanosheets from in situ topotactic transformation for selective electrocatalytic CO_2 reduction to formate. *Nat. Commun.* **9,** 1320 (2018).

19. Luc, W. et al. Ag-Sn Bimetallic Catalyst with a Core-Shell Structure for CO_2 Reduction. *J. Am. Chem. Soc.* **139,** 1885-1893 (2017).

20. Lei, F. et al. Metallic tin quantum sheets confined in graphene toward high-efficiency carbon dioxide electroreduction. *Nat. Commun.* **7,** 12697 (2016).

21. He, S. et al. The p-Orbital Delocalization of Main-Group Metals to Boost CO_2 Electroreduction. *Angew. Chem. Int. Ed. Engl.* **57,** 16114-16119 (2018).

22. Li, F. et al. Hierarchical Mesoporous SnO_2 Nanosheets on Carbon Cloth: A Robust and Flexible Electrocatalyst for CO_2 Reduction with High Efficiency and Selectivity. *Angew. Chem. Int. Ed. Engl.* **56,** 505-509 (2017).

23. Xia, C. et al. Continuous production of pure liquid fuel solutions via electrocatalytic CO_2 reduction using solid-electrolyte devices. *Nat. Energy* **4,** 776-785 (2019).

24. Ye, K. et al. In Situ Reconstruction of a Hierarchical Sn-Cu/SnO_x Core/Shell Catalyst for High-Performance CO_2 Electroreduction. *Angew. Chem. Int. Ed.*

Engl. **59,** 4814-4821 (2020).

25. Wen, G. et al. Orbital Interactions in Bi-Sn Bimetallic Electrocatalysts for Highly Selective Electrochemical CO_2 Reduction toward Formate Production. *Adv. Energy Mater.* **8,** 1802427 (2018).
26. Tang, J. et al. Advantages of eutectic alloys for creating catalysts in the realm of nanotechnology-enabled metallurgy. *Nat. Commun.* **10,** 4645 (2019).
27. Xing, Y. et al. Bi@Sn Core-Shell Structure with Compressive Strain Boosts the Electroreduction of CO_2 into Formic Acid. *Adv. Sci.* **7,** 1902989 (2020).
28. Yang, Q. et al. Novel Bi-Doped Amorphous SnO_x Nanoshells for Efficient Electrochemical CO_2 Reduction into Formate at Low Overpotentials. *Adv. Mater.* **32,** 2002822 (2020).
